# Rotaxane-catalyzed aerobic oxidation of primary alcohols

**DOI:** 10.1038/s42004-024-01375-0

**Published:** 2024-11-27

**Authors:** Ilario Baù, Cecilia Poderi, Francesca Sardu, Alessia Giancola, Anna Turchetti, Paola Franchi, Lorenzo Casimiro, Leonardo Andreoni, Serena Silvi, Elisabetta Mezzina, Marco Lucarini

**Affiliations:** 1 Department of Chemistry “Giacomo Ciamician”, University of Bologna, Via P. Gobetti 85, I-40129 Bologna, Italy; 2 Department of Industrial Chemistry “Toso Montanari”, University of Bologna, Via P. Gobetti 85, I-40129 Bologna, Italy; 3 Center for Light Activated Nanostructures, Istituto per la Sintesi Organica e la Fotoreattività, Consiglio Nazionale delle Ricerche via P. Gobetti 101, I-40129 Bologna, Italy; 4 Present Address: Sorbonne Université, CNRS, Institut Parisien de Chimie Moléculaire, IPCM, F-75005 Paris, France

**Keywords:** Interlocked molecules, Organocatalysis

## Abstract

Nitroxide radicals are widely utilized as catalysts for the oxidation of primary alcohols. Here, the aerobic catalytic oxidation cycle of nitroxide radicals has been implemented within a mechanically interlocked rotaxane architecture consisting of a paramagnetic crown ether, which is confined by a molecular axle containing a dialkylammonium station and a 1,2,3-triazole unit. The rotaxane is engineered to exploit the oxidation of a primary alcohol: the primary catalyst is the wheel, a nitroxide radical capable of altering its oxidation state during the catalytic cycle, while the co-oxidant is the Cerium(IV)/O_2_ couple. The synthesis of the proposed rotaxane, along with its characterization using EPR, HRMS, voltammetry and NMR data, is reported in the paper. The aerobic catalytic oxidation cycle was further investigated using EPR, NMR and GC-MS analyses. This study can aid in the design of autonomously driven molecular machines that exploit the aerobic catalytic oxidation of nitroxide radicals.

## Abstract (web summary)

Catalytic cycles have been demonstrated in mechanically interlocked systems. Here, the authors report a [2]rotaxane containing a nitroxidic radical macrocycle and establish the efficiency of its catalytic redox cycle in this constrained environment.

## Introduction

In recent decades, numerous examples of synthetic molecular machines^1^ activated by various stimuli have emerged in the literature. These artificial molecular machines are often mechanically interlocked compounds, such as rotaxanes and catenanes, held together by mechanical rather than chemical bonds. Inspired by the intricacies of complex biological systems, these molecules are engineered with precise recognition sites. When favorable interactions between their components are present, they form well-defined states at equilibrium. If an external energy source—such as light^2,3^, electrochemical input^4^, modulation of the chemical environment, or a catalytic reaction—drives the system away from equilibrium, a new state is produced by the displacement of one interlocked component with respect to the other.

However, the shuttling movement in most of the molecular machines still relies on a single stimulus to shift the machine into a new state, requiring manual addition of a counter-stimulus to reset the system to its original state^5–9^. As an example, in the case of molecular switches, acid–base chemistry is commonly employed to induce shuttling motions through the sequential addition of acidic and basic species^10–12^.

In 2016, Leigh and co-workers described a [2]-catenane in which a small molecular wheel was continuously transported directionally via a ratchet mechanism around a larger macrocyclic structure^13^. This was achieved through the step-by-step attachment and cleavage of a 9-fluorenylmethoxycarbonyl group over the large macrocycle. In this assembly, the directional rotation of the molecular motor persisted as long as the reactant, fluorenylmethoxycarbonyl chloride was present. The chemical species consumed to power the chemical engine was termed ‘fuel’^14^, a term that has been adopted in several subsequent examples.

Afterward, Di Stefano and coworkers described^15–17^ the use of 2-cyano-2-phenylpropanoic acid, leveraging the decarboxylation property of the acid to provide both a proton source (stimulus) and a proton acceptor (counter-stimulus). This allowed the activation of a complete cycle of motion in a Sauvage-type catenane^15^. The decarboxylation methodology was later applied to investigate the shuttling movement of a nitroxide-incorporating crown ether wheel^18^ in a paramagnetic [2]-rotaxane using EPR spectroscopy^19^.

The use of acids undergoing decarboxylation was further extended to trichloroacetic acid by Leigh and coworkers^20^, enabling one of the macrocycles in a [2]-catenane to perform a 360° circumrotation in one direction around the other. A [2]rotaxane molecular shuttle containing secondary ammonium/amine and thiourea stations that can be switched between catalytically active and inactive states using pulses of a chemical fuel has also been recently reported^21^. Quite recently, Stoddart et al. proposed a redox-driven rotary motor based on a [3]catenane in which two rings can be powered by electricity to rotate unidirectionally around a loop^22^.

In the current study, we report the investigation of a [2]rotaxane containing a nitroxidic radical macrocycle and its catalytic activity, which could be potentially exploited to trigger the motion of the macrocycle.

**Fig. 1:**
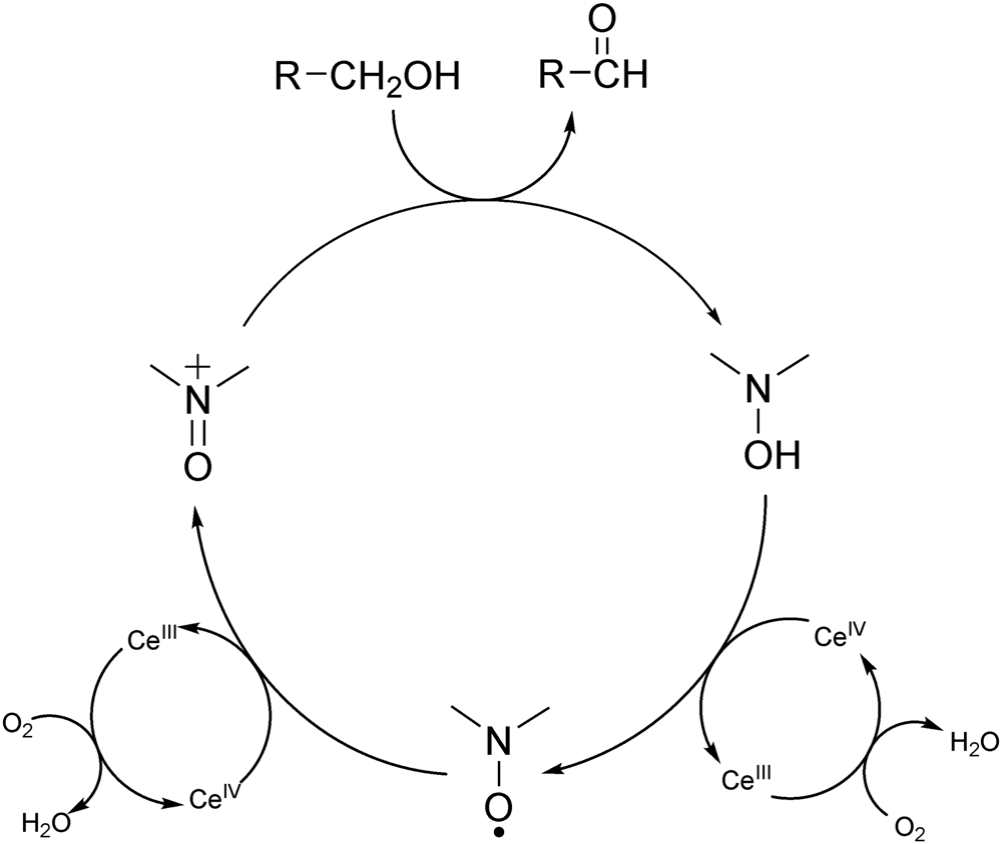
Catalytic cycle of the oxidation of primary alcohols promoted by nitroxide and cerium (IV) ammonium nitrate (CAN), in presence of oxygen. The oxoammonium cation, formed in situ by the oxidation of nitroxide, catalyzes the oxidation of the alcohol to aldehyde, forming the corresponding hydroxylamine. This is re-oxidized to nitroxide thanks to two equivalents of CAN, which in turn is continuously regenerated by oxygen.

Actually, the nitroxide radicals^23^ have been extensively studied in catalytic redox systems^24–27^ coupled to a secondary oxidant. For example, 2,2,6,6-tetramethylpiperidinyl-1-oxyl (TEMPO) radical has been frequently employed as a catalyst for the mild and selective oxidation of alcohols. *N*-oxoammonium salt can be generated in a catalytic way^28–33^ in situ by reacting TEMPO with numerous oxidants. The use of oxygen as a primary oxidant in conjunction with TEMPO and a transition metal catalyst (aerobic oxidation) has been investigated by a number of researchers. In general, the alcohol is oxidized by the oxoammonium cation, giving the corresponding N-OH amine (TEMPOH) as a co-product. The metal catalyst oxidizes the hydroxylamine to TEMPO, which is further converted by a second equivalent of the metal catalyst back to the key oxoammonium salt. The reduced form of the metal catalyst is recovered to the active form by oxygen as the primary oxidant (see Fig. 1).

Oxidation reaction catalyzed by TEMPO radicals, in combination with a secondary reaction involving iodosylbenzene and acetic acid, has been utilized to promote the continuous back-and-forth motion of the wheel in a switchable helicarene-based molecular shuttle^34^. However no reports describing nitroxide functionality mechanically trapped in a rotaxane structure still able to perform catalytic activity have been reported so far in the literature.

**Fig. 2:**
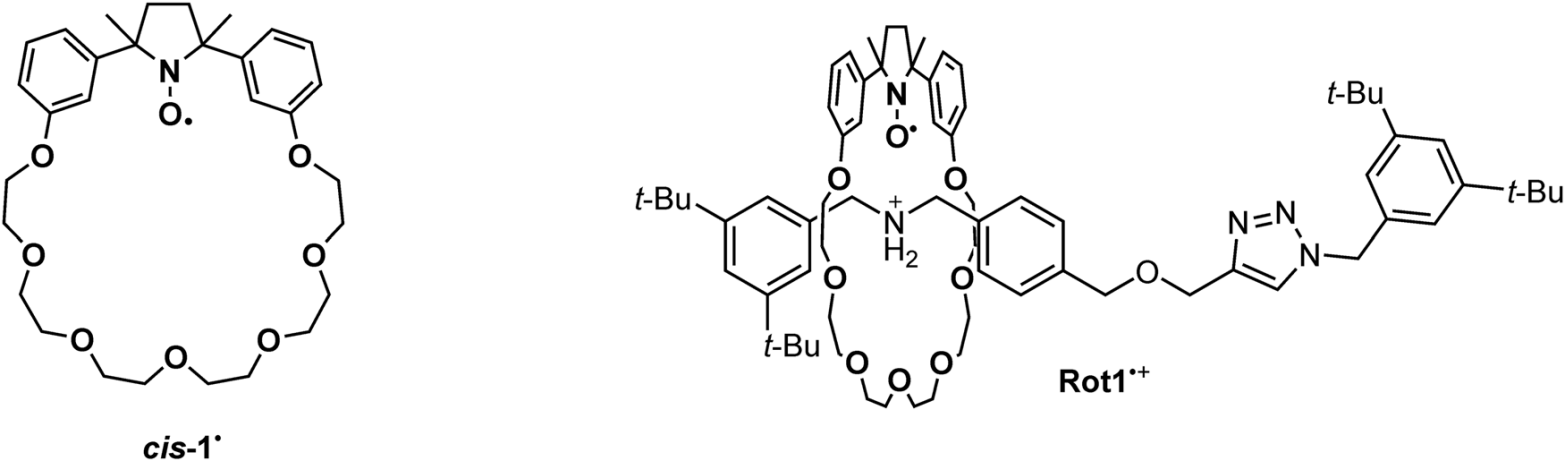
Structures of the nitroxides investigated in the present study. Macrocycle *cis*-**1**^**•**^ and rotaxane **Rot1**^•+^.

Here, we demonstrate the feasibility of an oxidative catalytic process within the constrained environment of a mechanically interlocked structure that possesses a nitroxidic radical character (Fig. 2). The proposed [2]-rotaxane (**Rot1**^•**+**^) is able to oxidize quantitatively primary alcohol to the corresponding aldehyde. Notably, the nitroxide macrocycle mechanically trapped in the molecular interlocked molecule during the oxidation process reversibly converts into the corresponding diamagnetic oxoammonium cation. This feature provides the foundations for the design of novel dissipative systems where the energy produced by the oxidation of primary alcohol to the corresponding aldehyde could be used to induce the motion of the macrocycle.

## Results and discussion

### Synthesis and characterization of Rot1^•^^+^

**Rot1**^•**+**^ consists of a paramagnetic crown ether, which is confined by a molecular axle containing a dialkylammonium station and a 1,2,3-triazole unit (see Fig. 2). As mentioned in the introduction, we have already reported a paramagnetic [2]-rotaxane containing a nitroxide-functionality incorporated in a crown ether wheel^18^. However, it was decided to synthesize and investigate a structure in which the original bis-pyridinium unit was replaced with a 1,2,3-triazole ring. Two main reasons led to this choice: the first is the easier synthesis due to the possibility of using a click reaction to perform rotaxanation^35^; the second is related to the potentially greater difficulty in oxidizing the nitroxide functionality to oxoammonium, owing to the presence of the two positive charges in the bis-pyridinium unit.

**Scheme 1:**
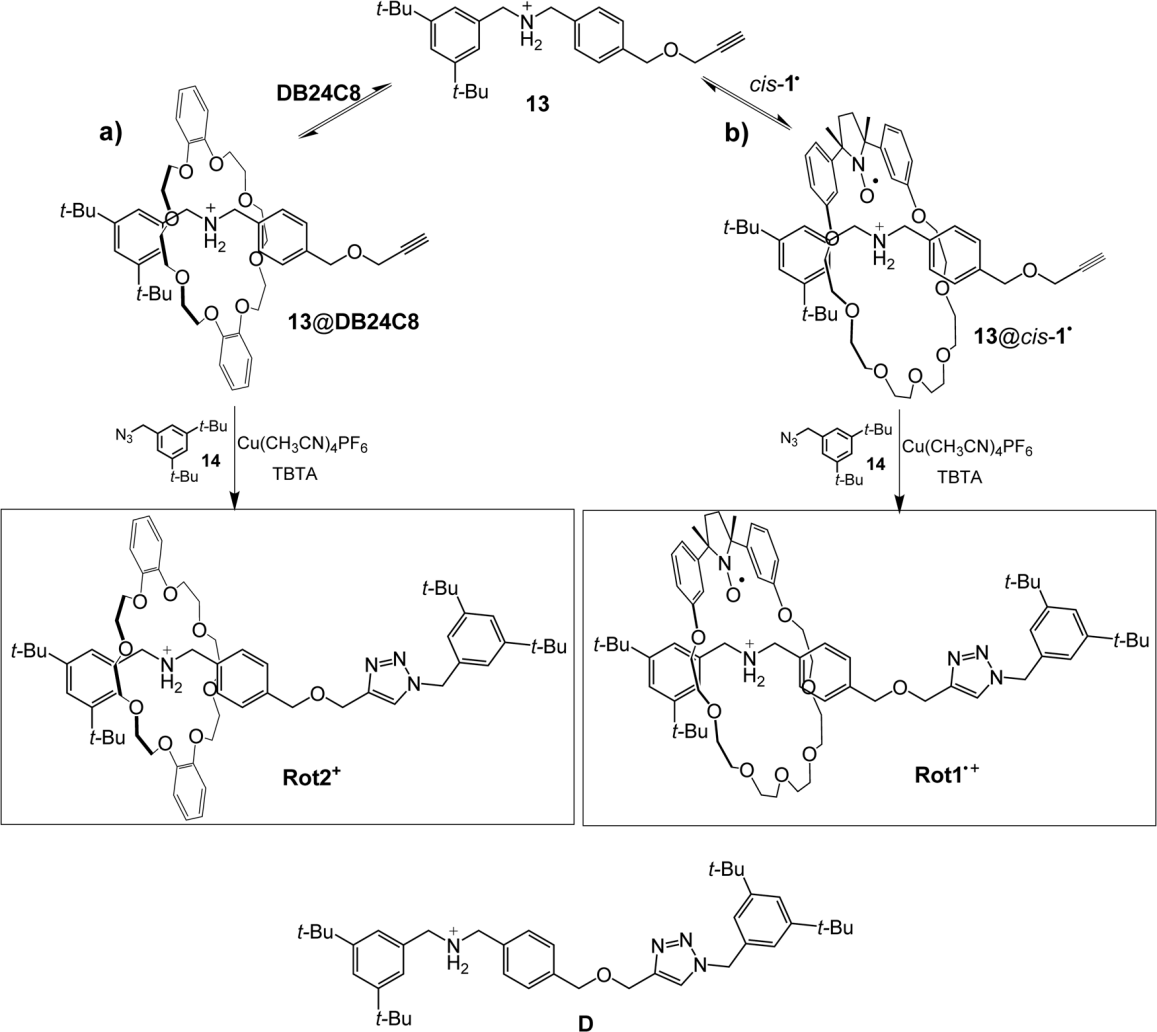
**Synthesis of the rotaxanes**. Synthetic route for the preparation of **Rot2**^**+**^ (**a**), **Rot1**^•+^ (**b**), and structure of the dumbbell **D**.

The target rotaxane was assembled using the *Stoddart threading-stoppering*^36^ approach, which involves complexation between the half-thread **13** and the crown ether, followed by interlocking with a suitable bulky stopper (see Scheme 1)^18,37^. In this process, the rotaxane formation is induced by the well-known Cu(I)-catalyzed *click* alkyne-azide cycloaddition (CuAAC)^38^, resulting in the formation of a 1,4-disubstituted triazole ring. Synthetic details concerning the preparation of half thread **13** and the macrocycle *cis*-**1**^**•**^ are reported in the Supplementary Methods. It should be noted that during the preparation of the paramagnetic rotaxane, the synthetic procedure for the macrocycle was improved compared to the original work^18^ (see Supplementary Methods).

Strong evidence for the formation of paramagnetic rotaxane was obtained by EPR spectroscopy. Using a known concentration of the radical TEMPO as a reference, EPR measurements indicated that the radical purity of the rotaxane was >95%.

**Fig. 3:**
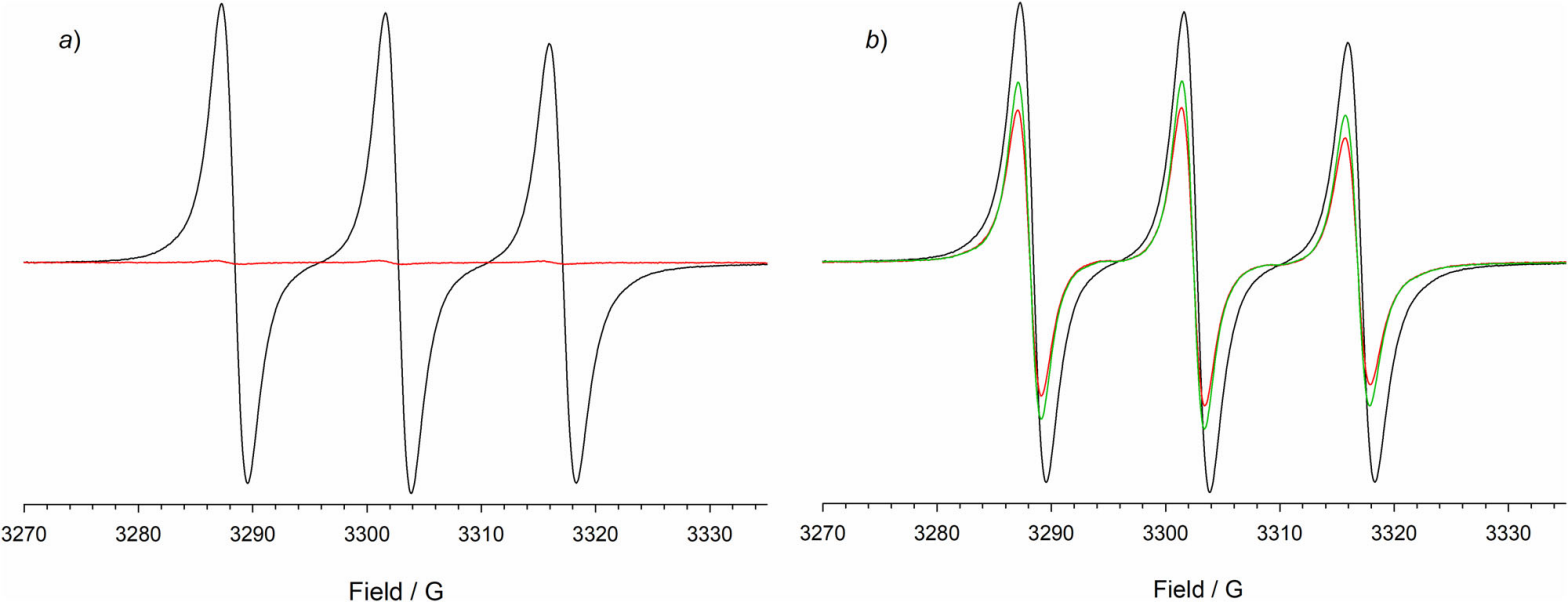
Oxidation of rotaxane **Rot1**^•+^ monitored by EPR. **a** EPR spectrum of **Rot1**^•+^ 1 mM in air-saturated acetonitrile, before (black line) and after (red line) the addition of 4 eq. of CAN. **b** EPR spectrum of **Rot1**^•+^ (1 mM) in air-saturated acetonitrile in the presence of *p*-methoxybenzyl alcohol (10 mM) before (black line) and after the addition of 4 eq. of CAN: red line *t* = 0, green line *t* = 1 hour.

The EPR spectrum of the rotaxane, recorded in CH_3_CN at 298 K, exhibits the typical three-line signal with the high-field line broadened due to restricted tumbling, and thus incomplete averaging of the anisotropic components of the hyperfine and g tensor (see Fig. 3, black line). This substantial reduction of the tumbling rate is typical for spin labels attached to larger molecules and can be considered as a further indication of the formation of the rotaxane. The measured value *a*_N_ = 14.39 G is very close to that of dibenzylammonium@*cis*-**1**^**•**^^18^, suggesting that the macrocycle is coordinating the dialkylammonium station of the thread.

This was further confirmed by the clear change in the nitrogen coupling observed upon adding one equivalent of a strong base, such as phosphazene P1-t-Bu, which is capable of deprotonating the NH_2_^+^ center, to a CH_3_CN solution of **Rot1**^**•+**^. Specifically, *a*_N_ value increases from 14.39 G to 14.63 G (see Supplementary Fig. 30).

Clear evidence for the formation of paramagnetic rotaxane was obtained also by ^1^H-NMR analysis. It is well known that NMR analysis of free radicals is not an easy task because the signals of paramagnetic compounds are broadened or sometimes not visible due to the efficient relaxation times of the protons near the radical^39,40^. Therefore, in an attempt to simplify NMR analysis, we decided to synthesize also a diamagnetic rotaxane analog (Scheme 1a) in which the paramagnetic wheel is replaced by commercially available dibenzo-24-crown-8 ether (**DB24C8**). For the synthetic details of the preparation of **Rot2**^**+**^ see SI.

**Fig. 4:**
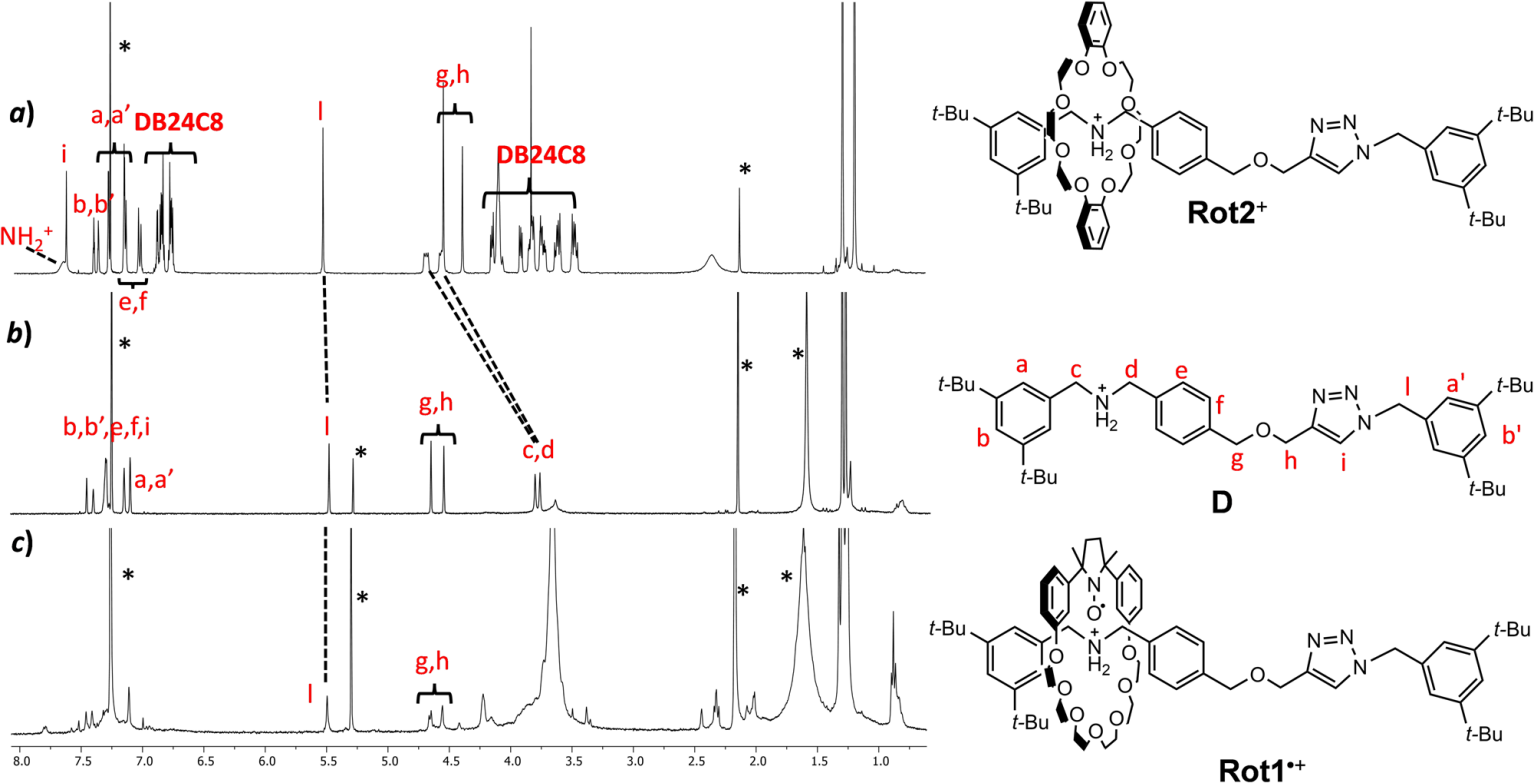
^1^H-NMR spectra in CDCl_3_ of the structures investigated in the present study. **a** Diamagnetic rotaxane **Rot2**^+^**;** **b**) dumbbell **D** and (**c**) radical rotaxane **Rot1**^•+^. Stars refer to the peaks of residual solvents. The signals are labeled according to the letters reported in the axle structure **D**.

Figure 4 compares the ^1^H-NMR spectra of the diamagnetic rotaxane (trace **a**) with that of the free molecular axle **D** (trace **b**). The spectrum of the rotaxane shows signals corresponding to both the dumbbell and the macrocycle. Notably, the proton peaks *g, h, i*, and *l* are indicative of the formation of the triazole ring in the axle component. The benzylic protons (*c* and *d*) adjacent to the ammonium station of the free axle **D** are significantly shifted downfield (indicated by dotted lines) when engaged in the rotaxane, indicating the position of the ring over the dialkylammonium site. The signals of the aromatic and polyether protons of **DB24C8** are splitted and well resolved due to the non-symmetric environment experienced by the locked crown ether nuclei.

In the ^1^H-NMR spectrum of the radical **Rot1**^**•+**^ (Fig. 4c), the signals of protons nearby the nitroxide moiety are either broadened or absent, as it happens to the benzylic CH_2_ ammonium signals *c* and *d* due to the proximity to the radical macrocycle *cis*-**1**^**•**^. The absence of these signals supports the EPR evidence that *cis*-**1**^**•**^ is predominantly located at the ammonium station. The absence of these signals confirms the EPR evidence that *cis*-**1**^**•**^ is preferentially situated at the ammonium station. This is confirmed also by the signals due to *g*, *h*, and *l* protons located in the triazole region, which appear at the same frequency as in the free dumbbell.

### Electrochemistry of *cis*-1^•^ and Rot1^•+^

**Fig. 5:**
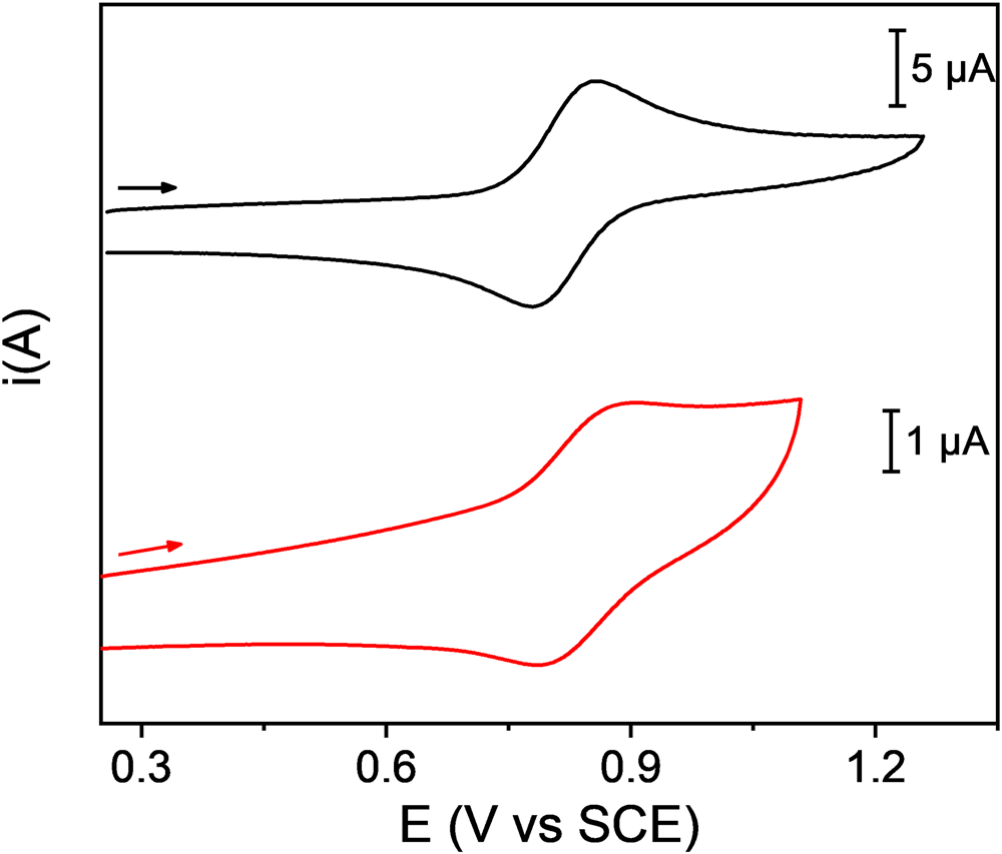
Cyclic voltammograms of the structures investigated in the present study. Cyclic voltammograms of a CH_3_CN solution of *cis****-*1**^**•**^ (black line, 5.0 × 10^−4^ M) and of **Rot1**^**•+**^ (red line, 1.5 × 10^−4^ M). Experimental conditions: argon-purged CH_3_CN, room temperature, 100 equivalents of TEAPF_6_, scan rate: 200 mV/s; decamethylferrocene (FcMe_10_) is used as internal standard.

The electrochemical properties of the cyclic nitroxide *cis*-**1**^**•**^ and of the rotaxane **Rot1**^**•+**^ were investigated by cyclic voltammetry (CV) in CH_3_CN and CH_2_Cl_2_ (Fig. 5 and Supplementary Fig. 29). For *cis*-**1**^**•**^, a reversible process, assigned to the oxidation of the nitroxide to the N-oxoammonium cation *cis*-**1**^**+**^, is observed at +0.82 V and +0.87 V vs SCE in CH_3_CN and in CH_2_Cl_2_, respectively. The different redox potentials in the two solvents can be attributed to the destabilization of the N-oxoammonium cation in the less polar CH_2_Cl_2_.

Rotaxane **Rot1**^**•+**^ is characterized by the same reversible process at +0.83 V and +0.88 V vs SCE in CH_3_CN and in CH_2_Cl_2_, respectively (Fig. 5 and Supplementary Fig. 29). As a matter of fact, the electrochemical properties of the cyclic nitroxide *cis*-**1**^**•**^ are not altered when the latter is incorporated in the rotaxane architecture; the process remains reversible and the redox potentials are only marginally affected.

### Macrocycle *cis*-1^•^ and rotaxane Rot1^•+^, catalyzed alcohol oxidation

**Scheme 2:**
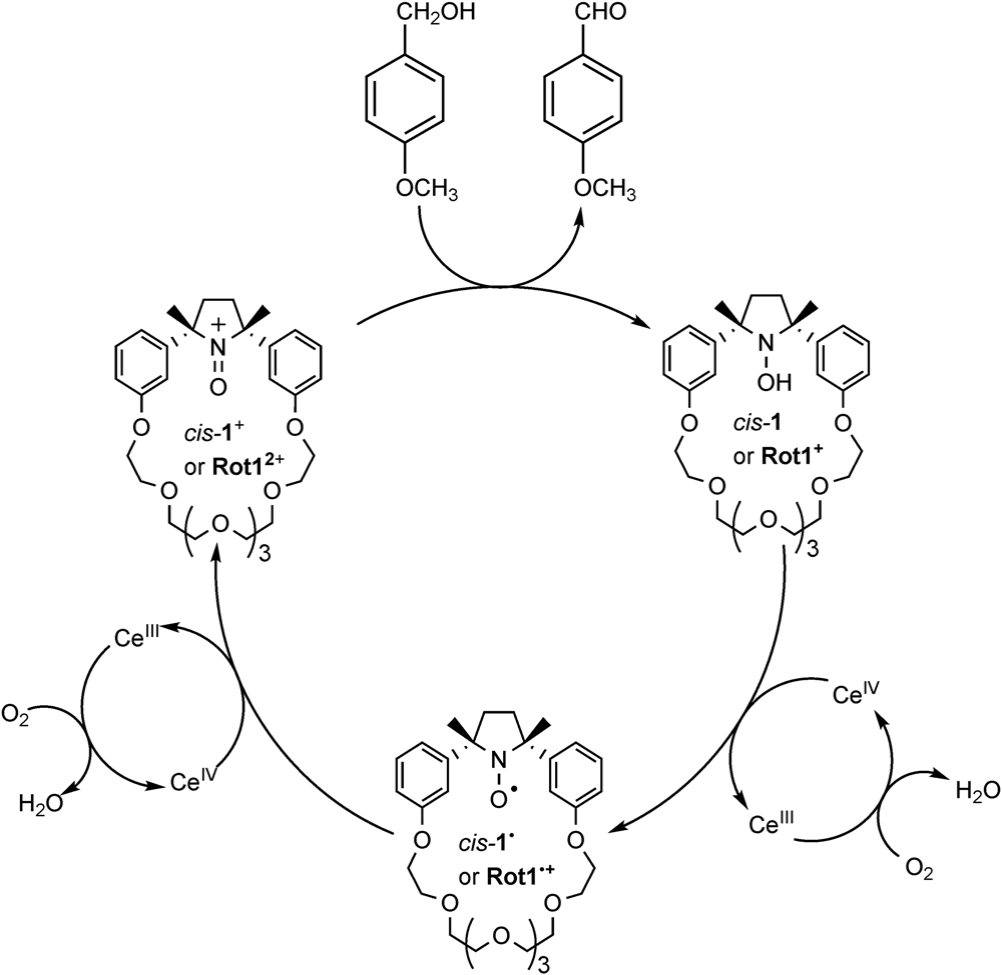
**Aerobic oxidation of a primary alcohol**. Oxidation of 4-methoxybenzyl alcohol to 4-methoxybenzaldehyde catalyzed by macrocycle *cis-***1**^**•**^ (or rotaxane **Rot1**^**•+**^) in the presence of CAN.

The efficiency of the paramagnetic crown ether *cis-***1**^***•***^ and rotaxane **Rot1**^**•+**^ in catalyzing the aerobic oxidation of primary alcohols to aldehydes using cerium(IV) ammonium nitrate (CAN) as a co-oxidant^33,41^ was then verified. Similar to the mechanism observed with the TEMPO radical, CAN converts the nitroxide into the oxoammonium cation, which is responsible for the oxidation of primary alcohols. CAN also regenerates the oxoammonium species from its reduced form (N-hydroxylamine), thereby sustaining a catalytic cycle (see Scheme 2)^33,41^.

GC–MS experiments were conducted to monitor the aerobic oxidation of 4-methoxybenzyl alcohol to 4-methoxybenzaldehyde using TEMPO as a catalyst and varying the amounts of CAN (20% or 40% relative to the initial alcohol concentration, Table 1 Entries 1–2). By monitoring the GC-MS chromatograms over time, the gradual disappearance of the alcohol peak was observed, together with the emergence of that of the aldehyde. The results showed increased conversion to aldehyde with higher CAN concentrations (up to 40% of the alcohol concentration). To unravel the role of oxygen in the catalytic cycle, the same experiments were conducted under an inert atmosphere (entries 3–4). Under these conditions, the conversions were significantly lower (see Table 1), as Ce(IV) could not be regenerated from Ce(III) in the absence of oxygen. The cessation of the catalytic cycle upon the consumption of all available Ce(IV) greatly reduced the reaction’s efficiency.

**Table 1:** Conversions calculated by GC–MS experiments performed on solutions containing 4-methoxybenzyl alcohol (10 mM), and nitroxide (1 mM) in CAN after 3 h of reaction

Entry	Nitroxide	[CAN]/mM	O_2_	Conversion/%
1	TEMPO	2	Air	70
2	TEMPO	4	Air	95
3	TEMPO	2	N_2_	10
4	TEMPO	4	N_2_	40
5	***cis-*1**^**•**^	2	Air	80
6	***cis-*1**^**•**^	4	Air	95
7	**Rot1**^**•+**^	2	Air	82^a^
8	**Rot1**^**•+**^	4	Air	97^a^
9	None	2	Air	8
10	None	4	Air	21

^a^Conversions determined by ^1^H-NMR analysis.

The reaction conditions optimized for the TEMPO radical were applied in the aerobic oxidation of 4-methoxybenzyl alcohol to 4-methoxybenzaldehyde using the nitroxide macrocycle *cis*-**1**^•^ as the catalyst (entries 5–6). Very good conversions of alcohol to aldehyde have been observed when *cis*-**1**^•^ was employed as a catalyst. The experimental results confirmed the occurrence of a catalytic cycle with *cis*-**1**^•^ ring and identified the optimal conditions for the complete oxidation of primary alcohol to aldehyde. As with the TEMPO radical, the yields were influenced by the amount of CAN co-catalyst, with the best conversion achieved using 40% CAN relative to the initial alcohol concentration.

NMR spectroscopy was instead employed to monitor the oxidation of 4-methoxybenzyl alcohol to 4-methoxybenzaldehyde using the rotaxane **Rot1**^**•+**^ as the catalyst (entries 7–8). The results showed excellent conversions, confirming the occurrence of a catalytic cycle even in the presence of the rotaxane.

Overall, these findings confirm that the excellent catalytic activity of the radical crown ether is maintained in the MIM, disclosing the potential of this catalytic cycle to fuel motion in molecular machines.

We also conducted GC–MS analysis of the oxidation in absence of the nitroxide radical (entries 9–10). The results revealed significantly lower conversion rates, indicating that CAN alone is not able to efficiently convert the alcohol to aldehyde, and the oxoammonium cation is required for sustaining efficiently the catalytic process.

### EPR investigation of *cis*-1^•^ and rotaxane Rot1^•^^+^, catalyzed alcohol oxidation

Once we demonstrated the ability of **Rot1**^**•+**^ to catalyze the aerobic oxidation of 4-methoxybenzyl alcohol to 4-methoxybenzaldehyde, we determined the oxidation state of the macrocycle via EPR analysis. This state is expected to vary during the catalytic cycle. Initially, the system is at equilibrium, with the nitroxide state of the cycle complexing the ammonium station (see Scheme 2). Upon addition of the alcohol, the macrocyclic nitroxide is continuously converted to the oxoammonium form (**Rot1**^2+^), which is then reduced back to nitroxide after reacting with the alcohol. This sequence of reactions switches the rotaxane between the two redox forms until the alcohol is depleted.

EPR analysis was conducted during the oxidation of rotaxane **Rot1**^**•+**^ (1 mM) in air-equilibrated CH_3_CN by adding 4 equivalents of CAN, both in the absence and presence of 10 equivalents of *p*-methoxybenzyl alcohol, and confirmed this hypothesis. In the first case (Fig. 3a), EPR measurements revealed that the **Rot1**^**•+**^ signal immediately disappeared due to the quantitative oxidation of the rotaxane nitroxide to oxoammonium. After 20, 40, and 60 minutes, and even after 60 h, the nitroxide signal was not recovered.

The same sequence of experiments was carried out in the presence of the alcohol (Fig. 3b). In this case, spectra were recorded at zero time and after 20, 40, and 60 min. The spectra in Fig. 3b show a less pronounced decrease in the EPR signal in the presence of alcohol after the addition of CAN. The amount of nitroxide fluctuates between 55% and 60% of the initial amount of **Rot1**^**•+**^.

These measurements clearly reflect the distribution of catalyst oxidation states during the reaction. According to the proposed mechanism^33^ (Scheme 2), there are three catalyst oxidation states: oxoammonium, nitroxide, and hydroxylamine species. It is generally assumed^42^ that the hydroxylamine, formed upon oxidation of the alcohol, undergoes rapid oxidation to the nitroxide either by direct reaction with the oxidant or via comproportionation with the oxoammonium species. Consequently, a negligible steady-state concentration of hydroxylamine is expected, and a near-quantitative mass balance between the oxoammonium and nitroxide species is maintained during the reaction. EPR measurements indicate that the predominant forms of the catalyst during steady-state turnover, nitroxide, and oxoammonium, are rather similar, with a slight excess of the former.

As an aerobic catalytic cycle, the presence of oxygen is essential to oxidize Cerium (III) to its active form Ce (IV). We verified this by repeating the EPR measurements in a closed tube containing **Rot1**^**•+**^, CAN, and the primary alcohol. Due to the Heisenberg spin exchange, the broadening of the EPR spectral lines of radicals is proportional to the concentration of oxygen dissolved in the solution. Thus, the change in oxygen concentration when the reaction is performed in a closed tube can be determined by measuring the line width of the three-line EPR spectrum of the rotaxane^43^. After a few hours, a significant narrowing of the radical linewidth was observed (from 2.3 G to 1.7 G), indicating an oxygen consumption similar to what occurs when working under an inert atmosphere.

The data strongly suggest that NO^•^ and N = O^+^ species serve as the primary transient forms of the catalyst during the oxidation cycle. Potentially, this could alter the macrocycle’s affinity for specific molecular stations, thereby facilitating a complete cycle of macrocycle movements. Even though in the present system there is no evidence of macrocycle movement, this study demonstrates the possibility of using the nitroxide-mediated oxidative catalytic cycle within the constrained environment of a mechanically interlocked molecule, with quantitative yields similar to those observed with TEMPO.

## Conclusions

This study opens up new perspectives for the design of dissipative systems, i.e., molecular structures that can be transiently varied upon the addition of a proper stimulus, called fuel, which is consumed over a given time.

To this aim, the synthesized rotaxane **Rot1**^•+^ was conceived to carry on the aerobic oxidation catalytic cycle of the nitroxide radical incorporated in the paramagnetic interlocked macrocycle in the presence of primary alcohol and of CAN/O_2_ as co-oxidants (Scheme 2). The MIM is composed of the radical macrocycle *cis-***1**^•^ and a dumbbell containing 1,2,3-triazole and dibenzylammonium ion as stations. The study provided the following results: (a) the addition of CAN causes a drastic decrease of the signal of the nitroxide radical, indicating the oxidation of nitroxide to a diamagnetic oxammonium cation of rotaxane; (b) after the addition of the primary alcohol the nitroxide signal reappears immediately, due to the triggering of the catalytic cycle of alcohol oxidation promoted by the oxammonium cation. The process continues autonomously until the alcohol is fully converted into the corresponding aldehyde. Despite the lack of shuttling, the current study can aid in the design of autonomously driven molecular machines that exploit the aerobic catalytic oxidation of nitroxide radicals.

## Methods

Details of all experimental procedures are provided in the Supporting Information.

## Supplementary information

Supporting Information

## Data Availability

The data analyzed and generated in this study are provided in Supplementary Information and the table included in this published article.
