# Supplementary material for: Rotaxane-catalyzed aerobic oxidation of primary alcohols
Source: Commun Chem. 2024 Nov 27;7:278. doi: 10.1038/s42004-024-01375-0 (PMC11603149; doi:10.1038/s42004-024-01375-0)
Supplement: Supplementary file 1 — Supporting Information [file 42004_2024_1375_MOESM1_ESM.pdf]

Supporting information for

Rotaxane-Catalyzed Aerobic Oxidation of Primary Alcohols

Ilario Baù, Cecilia Poderi, Francesca Sardu, Alessia Giancola, Anna Turchetti, Paola Franchi, Lorenzo Casimiro, Leonardo Andreoni, Serena Silvi, Elisabetta Mezzina & Marco Lucarini

---

1. Supplementary Methods
- 1.1 General Information 2
- 1.2 General procedure for the synthesis of the radical crown ether *cis*-**1•**. 2
- 1.3 Synthesis of 2-(3-methoxyphenyl)-2-methylpyrrolidin-1-ol (**2b**) 3
- 1.4 Synthesis of 2-(3-methoxyphenyl)-2-methyl-3,4-dihydro-2H-pyrrole-1-oxide (**3**) 4
- 1.5 Synthesis of 2,5-bis(3-methoxyphenyl)-2-methyl-3,4-dihydro-2H-pyrrole 1-oxide (**4**) 4
- 1.6 Synthesis of *cis*-2,5-bis(3-methoxyphenyl)-2,5-dimethylpyrrolidin-1-oxyl (*cis*-**5•**) and *trans*-2,5-bis(3-me-thoxyphenyl)-2,5-dimethylpyrrolidin-1-ol (*trans*-**5**) 5
- 1.7 Synthesis of 3,3'-(1-hydroxy-2,5-dimethylpyrrolidine-2,5-diyl)diphenol (*cis*-**6**) 6
- 1.8 Synthesis of macrocycle *cis*-**1** 6
- 1.9 General procedure for the synthesis of alkyne **13** 7
- 1.10 Synthesis of methyl 4-(((*tert*-butoxycarbonyl)amino)methyl)benzoate (**8**) 7
- 1.11 Synthesis of *tert*-butyl(4-(hydroxymethyl)benzyl)carbamate (**9**) 8
- 1.12 Synthesis of *tert*-butyl (4-((prop-2-yn-1-yloxy)methyl)benzyl)carbamate (**10**) 8
- 1.13 Synthesis of (4-((prop-2-yn-1-yloxy)methyl)phenyl)methanamine (**11**) 9
- 1.14 Synthesis of alkyne **13** 9
- 1.15 Synthesis of 1-(azidomethyl)-3,5-di-*tert*-butylbenzene (**14**) 10
- 1.16 Synthesis of dumbbell **D** 10
- 1.17 Synthesis of Rotaxane (**Rot2^{+}**) 11
- 1.18 Synthesis of Rotaxane (**Rot1$^{+•}$**) 11
- 1.19 GC-MS measurements 12
- 1.20 Electrochemical measurements 12
- 1.21 ESR Measurements of **Rot1**$^{+•}$ in the presence of a base 13

2. Supplementary Figures 14

3. Supplementary References 31

---

1. Supplementary Methods

1.1 General information

EPR spectra has been recorded on Bruker-ELEXYS spectrometer by using the following instrument settings: microwave power 0.79 mW, modulation amplitude 0.04 mT, modulation frequency 100 kHz, scan time 180 s, 2K data points. The hyperfine splittings were determined by computer simulation using a Monte Carlo minimisation procedure.[1]

^{1}H NMR spectra of the rotaxanes **Rot2**$^{+}$ and **Rot1**$^{\bullet+}$, the dumbbell **D** and macrocycle *cis*-**1**$^{\bullet}$ together with its precursors were recorded at 298 K on a Varian Mercury spectrometer operating at 400 MHz in $CDCl_3$ and CD_3SOCD_3 solutions using the solvent peak as internal standard (7.26 and 2.50 ppm). ^{13}C-NMR spectra of the rotaxane **Rot2**$^{+}$, the dumbbell **D** together with its precursors were recorded at 298 K on a Varian Mercury spectrometer operating at 100 MHz. in $CDCl_3$ solutions using the solvent peak as internal standard (77.0 ppm). Chemical shifts are reported in parts per million (δ scale).

HRMS spectra of the the rotaxanes **Rot2**$^{+}$, **Rot1**$^{\bullet+}$, and macrocycle *cis*-**1**$^{\bullet}$ were recorded on Waters Xevo G2-XS QTof by using the following instrumental settings: positive ion mode; desolvation temp. 600° C; capillary voltage: 0.8 kV; cone voltage: 30 V. (Cone gas flow: 50 L/h desolvation gas flow: 1000 L/h).

All reagents were used without further purification and are commercially available. Reaction solvents were bought dry and used directly.

1.2 General procedure for the synthesis of the radical crown ether *cis*-1$^{\bullet}$.

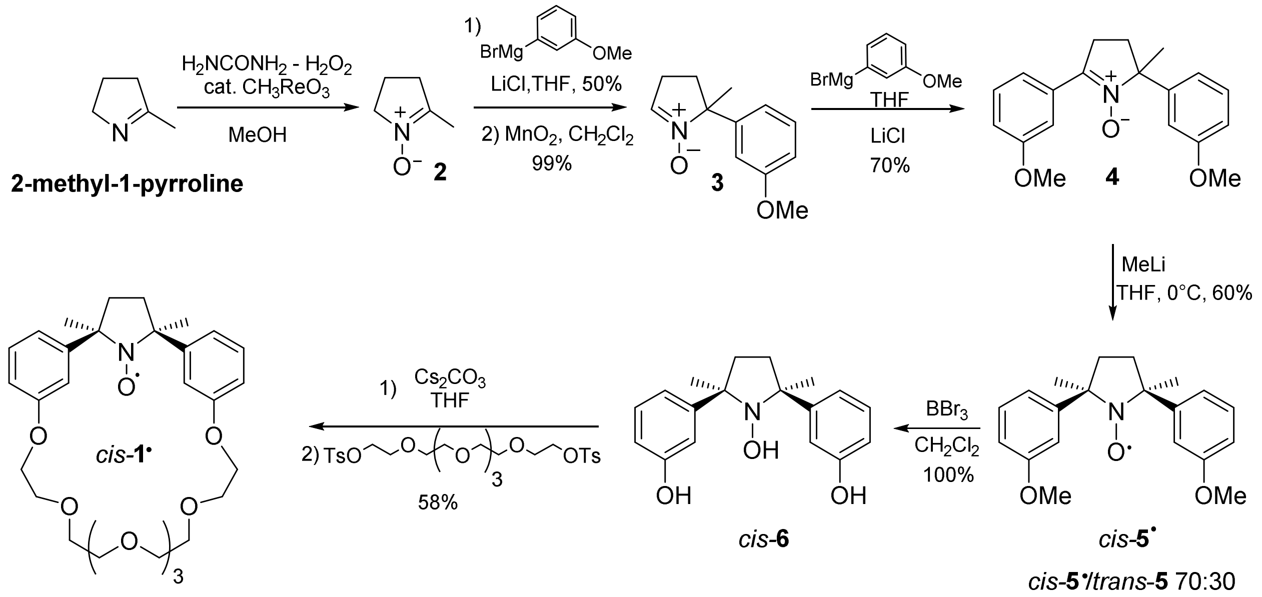

Crown ether *cis*-**1˙** was prepared following an improved procedure compared to what was previously reported.[1] The first three steps are modifications of previously described methods,[1] the reaction sequence was enhanced by adding MeLi to the key intermediate nitrone **4**, producing a mixture of isomers with a preference for the *cis*-N-hydroxy pyrrolidine. Chromatographic separation of the crude mixture easily yields *cis*-**5˙** in its radical form, thereby preventing the oxidation step of the N-OH derivative to nitroxide. The ability to work with a pure isomer, eliminating the need to separate the final nitroxide products as required in the previous synthesis,[1] represents a further improvement in this new procedure and increases the final paramagnetic macrocycle yield. The isolated *cis* isomer was then demethylated by BBr_3 yielding the diphenol *cis*-**6**. This compound underwent cyclization using a bis-tosylated glycole chain to give the desired macrocycle *cis*-**1˙**.

1.3 Synthesis of 2-(3-methoxyphenyl)-2-methylpyrrolidin-1-ol (2b)

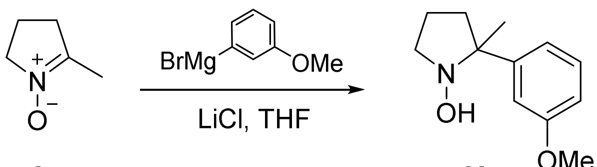

2 **2b**

To a solution of 3-Methoxyphenylmagnesium bromide 1 M in THF (15 mL) was added a solution of dry LiCl (1M, 1 eq) in anhydrous THF. Then nitrone **2** (1 g, 10 mmol) dissolved in anhydrous THF (20 mL) was added dropwise at 0 °C over a period of 30 min using a syringe pump. The reaction mixture was stirred for 2 h and after this period was quenched by addition of a saturated solution of NH_4Cl. The aqueous layer was extracted with ethyl acetate (AcOEt) and then the combined organic extracts were washed with water and brine, dried over $MgSO_4$, filtered, and concentrated under vacuum. The remaining oil was used in the successive step without purification. One portion of the crude was purified by silica gel column (cyclohexane/AcOEt 6:4) giving compound **2b** (Rf=0.46) in 50% yield. The ^{1}H-NMR spectrum was comparable to that of **2b** previously obtained.[1]

^{1}H NMR (400 MHz, $CDCl_3$): δ 1.48 (s, 3H, CH_3), 1.92-2.10 (m, 3H, CH), 2.29-2.38 (m, 1H, CH), 3.25-3.33 (m, 2H, CH_2), 3.82 (s, 3H, OCH_3), 6.80 (ddd, J = 8.0, 2.4 e 0.8 Hz, 1H, Ar), 7.09 (ddd, J = 8.0, 1.6, 0.8 Hz, 1H, Ar), 7.13 (dd, J = 2.4 e 1.6 Hz, 1H, Ar), 7.26 (t, J = 8 Hz, 1H, Ar) ppm.

^{13}C NMR (100 MHz, $CDCl_3$): δ 20.4, 25.0, 29.7, 37.0, 55.2, 79.3, 111.5, 112.4, 118.5, 129.2, 147.7, 159.8 ppm.

1.4 Synthesis of 2-(3-methoxyphenyl)-2-methyl-3,4-dihydro-2H-pyrrole-1-oxide (3)

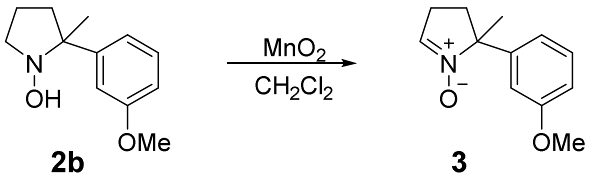

To a solution of compound **2b** (0.600 g, 2.89 mmol) in CH_2Cl_2 (6 mL) 1.5 equivalents of MnO_2 were added at 0°C, and the reaction was stirred for 2 hs at room temperature. After filtration over Celite and sodium sulfate, and evaporation under vacuo the crude (brown oil) was used without further purification resulting the desired product **3** as confirmed by ^{1}H-NMR spectrum in 99% yield.[2] The ^{1}H-NMR spectrum matches that of **3** previously obtained.[1]

^{1}H NMR (400 MHz, $CDCl_3$): δ 1.85 (s, 3H, CH_3), 2.32-2.42 (m, 1H, CH), 2.50-2.64 (m, 3H, CH, CH_2), 3.81 (s, 3H, OCH_3), 6.83 (dd, *J* = 8.0, 2.0 Hz, 1H, Ar), 6.91-6.98 (m, 2H, Ar, CH), 7.06 (*br*s, 1H, Ar), 7.28 (t, *J* = 8.0 Hz, 1H, Ar) ppm.

^{13}C NMR (100 MHz, $CDCl_3$): δ 24.9, 25.0, 37.0, 55.2, 79.3, 111.7, 112.6, 117.5, 129.7, 134.2, 143.0, 159.8 ppm.

1.5 Synthesis of 2,5-bis(3-methoxyphenyl)-2-methyl-3,4-dihydro-2H-pyrrole 1-oxide (4).

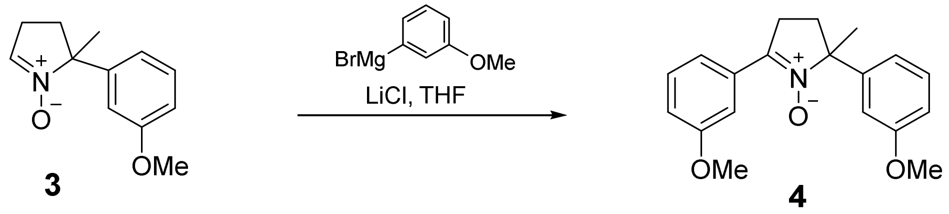

To a solution of 3-Methoxyphenylmagnesium bromide 1 M in THF (1.5 eq, 5.85 mL) was added a solution of dry LiCl (1 M, 1 eq) in anhydrous THF. Then nitrone **3** (0.8 g, 3.9 mmol) dissolved in anhydrous THF (10 mL) was added dropwise at 0 °C over a period of 30 min using a syringe pump. The reaction mixture was stirred for 5 hs and after this period was evaporated under vacuum. The residue was treated with 0.5 M HCl and the aqueous layer was extracted with AcOEt. The combined organic extracts were washed with water and brine, dried over $MgSO_4$, filtered, and concentrated under vacuum. The crude was purified using silica gel column (cyclohexane/AcOEt 6:4, Rf=0.34) giving exclusively nitrone **4** in 30% yield (1.17 mmol, 0.36 g). In this case the oxidation reaction of the intermediate N-hydroxylamine (see the corresponding reaction **2b→3**) to nitrone was not necessary.

^{1}H NMR (400 MHz, $CDCl_3$): δ 1.94 (s, 3H, CH_3), 2.35 (dt, J =12.8, 9.0 Hz, 1H, CH), 2.54 (ddd, J =12.8, 8.5, 3.0 Hz, 1H, CH), 3.03 (heptet, J = 8.5 Hz, 1H, CH), 3.10 (ddd, J = 16.7, 9.0 e 3.0 Hz 1H, CH), 3.78 (s, 3H, OCH_3), 3.88 (s, 3H, OCH_3), 6.81 (ddd, J = 8.0, 2.7 e 1.0 Hz, 1H, Ar), 6.89-6.93 (m,

2H, Ar), 7.02 (ddd, J = 8.0, 2.7 e 1.0 Hz, 1H), 7.26 (t, J = 8.0 Hz, 1H, Ar), 7.36 (t, J = 8.0 Hz, 1H, Ar), 7.65 (ddd, J = 8.0, 1.6, 1.0 Hz,1H, Ar), 8.52 (dd, J = 2.7, 1.5 Hz 1H, Ar) ppm.

^{13}C NMR (100 MHz, $CDCl_3$): δ 25.5, 27.5, 36.6, 55.2, 55.3, 81.6, 111.5, 111.9, 112.2, 117.3, 117.6, 120.1, 129.2, 129.4, 129.7, 130.7, 144.0, 159.5, 159.8 ppm.

1.6 Synthesis of cis-2,5-bis(3-methoxyphenyl)-2,5-dimethylpyrrolidin-1-oxyl (*cis*-5•) and trans-2,5-bis(3-methoxyphenyl)-2,5-dimethylpyrrolidin-1-ol (*trans*-5).

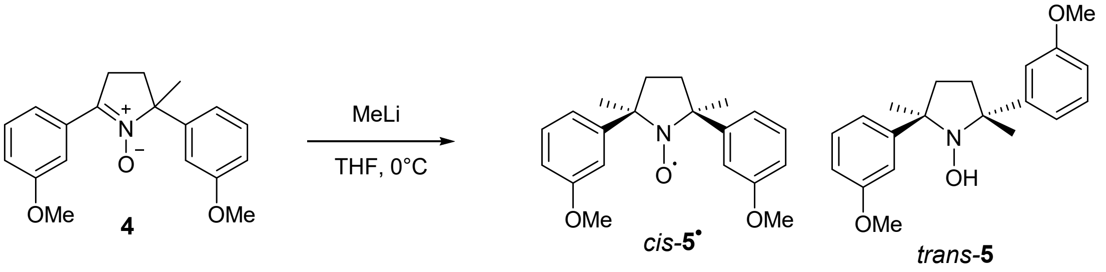

cis-**5**•/*trans*-**5**
70:30

To a 20 mM solution of **4** (0.047 g, 0.15 mmol) in anhydrous THF (7.5 mL) at 0°C and under stirring, a solution of 1.6 M MeLi in Et_2O (0.19 mL, 0.30 mmol) is added under nitrogen atmosphere. The reaction is allowed to proceed for 30 minutes and followed by TLC (cyclohexane:AcOEt=6:4) and GC-MS analysis. Both the analyses reveled the presence of two isomers, presumably *cis*-**5** and *trans*-**5** hydroxylamines. The former is the main product of the reaction at 0°C (94:6 *cis/trans* ratio). After evaporation of the solvent a saturated solution of NH_4Cl was added. The aqueous layer was extracted with CH_2Cl_2 and the combined organic extracts dried over Na_2SO_4, filtered, and concentrated under vacuum. The crude was purified by silica gel column eluting with cyclohexane:AcOEt=8:2. During the work-up procedure compound *cis*-**5** oxidizes to the corresponding nitroxide and was recovered exclusively in the radical form *cis*-**5**• (Rf = 0.55, 0.03 g, 0.091 mmol) in 60% yield. This behaviour is not new for N-hydroxy derivatives that undergo redox processes more efficiently for *cis* than for *trans* isomer because of the lower steric hindrance around the N-OH functionality in the former one.[1]

^{1}H NMR (400 MHz, $CDCl_3$) (*cis*-**5**•): δ 3.83 (s, OCH_3), 7.36 (*br*, Ar).
EPR (CH_2Cl_2) (*cis*-**5**•): a_N=14.48 G, g=2.0062.

^{1}H NMR (400 MHz, $CDCl_3$) (*trans*-**5**): δ 1.68 (s, 6H, CH_3), 1.97-2.14 (m, 4H, CH_2), 3.83 (s, 6H, OCH_3), 6.77 (ddd, J = 8.0, 2.6 and 1.1 Hz, 2H, Ar), 7.19 (dt, J = 7.7 and 1.5 Hz, 2H, Ar), 7.24 (t, 2H, J = 1.5 Hz, Ar), 7.28 (t, 2H, J = 8.0 Hz, Ar) ppm.

^{13}C NMR (100 MHz, $CDCl_3$) (*trans*-**5**): δ 22.0, 37.1, 54.4, 67.7, 109.9, 111.4, 117.4, 128.2, 151.0, 158.6 ppm.

1.7 Synthesis of 3,3'-(1-hydroxy-2,5-dimethylpyrrolidine-2,5-diyl)diphenol (*cis*-6)

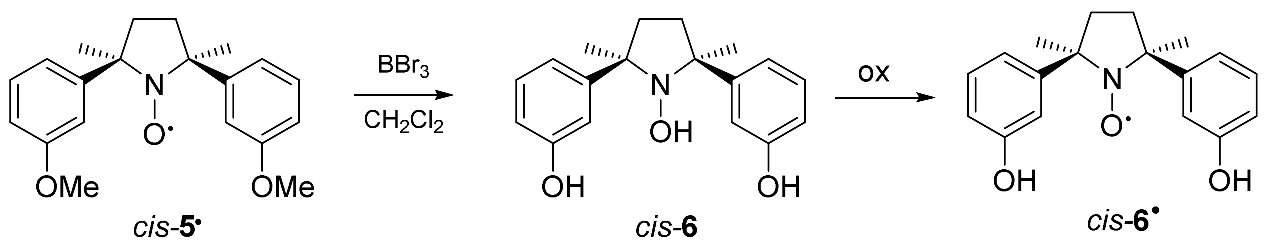

To a stirred 0.1 M solution of *cis*-**5•** (0.03 g, 0.091 mmol) in CH_2Cl_2 at 0°C, was added dropwise a fresh 1 M solution of BBr_3 (0.23 mL, 2.5 eq) in dichloromethane. The reaction was monitored by TLC (cyclohexane:AcOEt=6:4, as eluent) and further BBr_3 solution was added, if necessary. After 30 min at 0 °C a mixture of ice and water was added. The organic phase is collected and the aqueous phase is extracted with AcOEt. The organic layers were dried over Na_2SO_4, and evaporated under vacuo. ^{1}H spectrum of the crude was acquired shortly after the work-up (see Supplementary Figure 9). Product *cis*-**6** (Rf = 0.45) resulted almost pure and was used in the next step without further purification. After few hours the N-hydroxylamine *cis*-**6** underwent oxidation to the corresponding nitroxide *cis*-**6•** as confirmed by EPR spectrum (reported below).

^{1}H NMR (400 MHz, d^6-DMSO) (*cis*-**6**): δ 1.48 (s, 6H), δ 1.70-1.80 (m, 2H, CH), 1.98-2.04 (m, 2H, CH), 6.54-6.59 (m, 2H, Ar), 7.00-7.11 (m, 6H, Ar), 7.28 (s, 1H, NOH), 9.12 (s, 2H, OH) ppm.

EPR (DMSO) (*cis*-**6•**): a_N=14.51 G, *g*=2.0059.

1.8 Synthesis of macrocycle *cis*-1

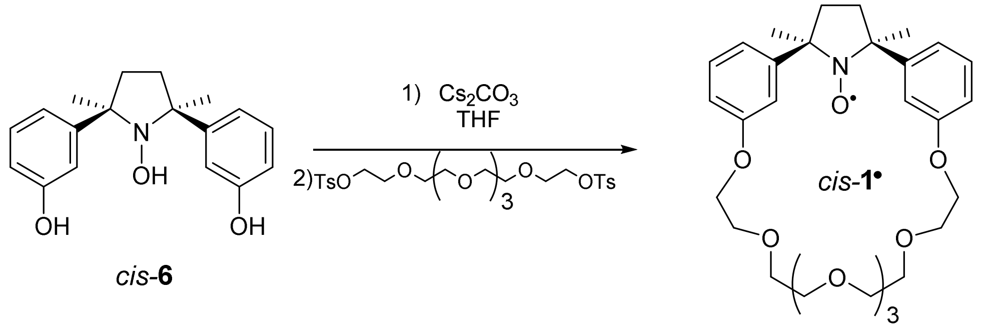

A solution 50 mM of cis-**6** (0.05 g, 0.167 mmol) in THF (3.5 mL) was added to a stirring suspension of Cs_2CO_3 (0.272 g, 0.835 mmol) in THF (8.35 mL) in a two necked round bottom flask under nitrogen atmosphere. After 15 minutes the mixture was diluted with 35 mL THF and treated with a 7.5 mM solution of ditosylate (0.099 mg, 0.167 mmol) by a syringe pump in 60 minutes. The final solution is pale pink.

The resulting mixture was heated at reflux for 12 hs. The solvent was evaporated in vacuum, water added and the aqueous layer was extracted with CH_2Cl_2. The extract was dried ($MgSO_4$), concentrated and purified by column chromatography eluting starting with CH_2Cl_2 until CH_2Cl_2/MeOH 95:5 to give compound *cis*-**1·** (0.053 g, 0.097 mmol) in 58% yield. The ^{1}H-NMR spectrum was comparable to that of *cis*-**1·** previously obtained.[1]

EPR (CH_2Cl_2): a_N=14.35 G, g=2.0061.

^{1}H NMR (400 MHz, $CDCl_3$): δ 3.50-4.20 (m, CH_2), 7.09-7.52 (m, Ar), 7.78-7.81 (m, Ar) ppm

1.9 General procedure for the synthesis of alkyne 13

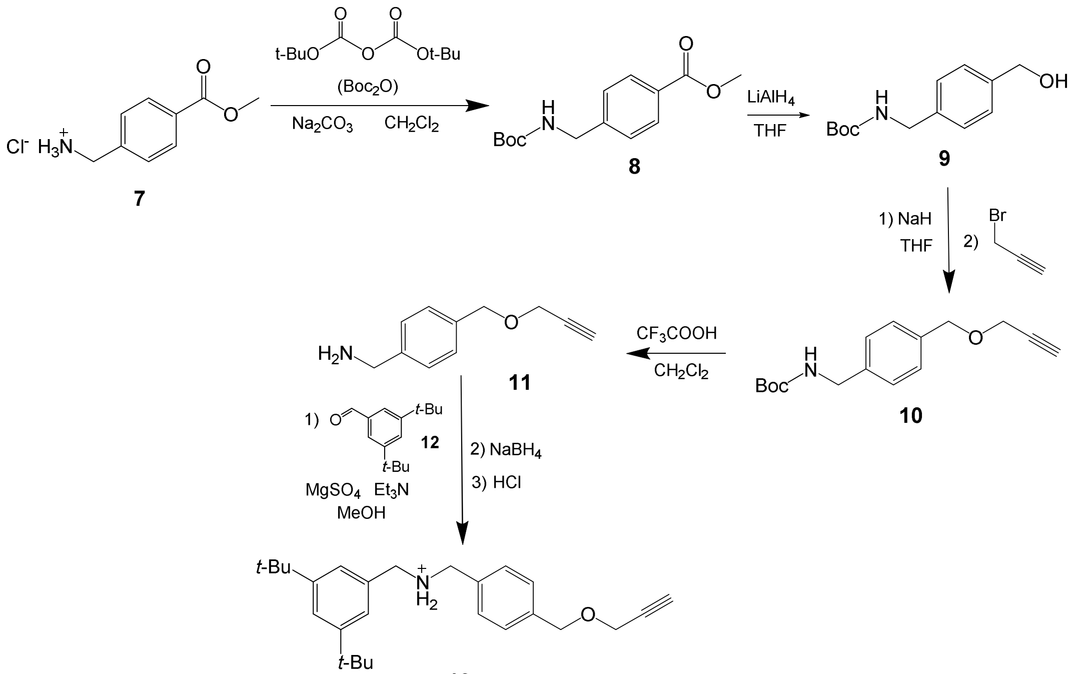

1.10 Synthesis of methyl 4-(((*tert*-butoxycarbonyl)amino)methyl)benzoate (8)

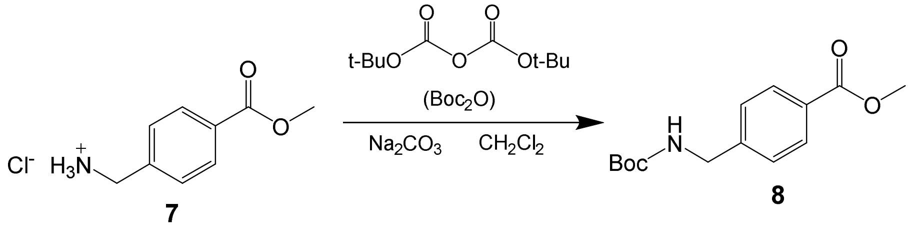

To a stirred solution of 50 mM of compound **7** (1 eq) and Na_2CO_3, di-*tert*-butildicarbonate (Boc_2O) (1 eq) was added at 0°C and the suspension was stirred overnight. After quenching with satured $NH_4^+Cl^-$, the aqueous phase was extracted with chloroform. The organic layer was dried over Na_2SO_4, and evaporated under vacuo. The protected amine **8** was obtained in 99% yield and used

without further purification in the next step. The spectroscopic data match those reported in the literature.[3]

^{1}H NMR (400 MHz, $CDCl_3$) δ 7.99 (d, J = 8.0 Hz, 2H, Ar), 7.34 (d, J = 8.0 Hz, 2H, Ar), 4.94 (br, 1H, NH), 4.36 (d, J = 5.9 Hz, 2H, CH_2), 3.90 (s, 3H, OCH_3), 1.45 (s, 9H, *t*-Bu) ppm.

^{13}C NMR (100.0 MHz, CDCl3) δ 169.9, 155.9, 144.2, 129.9, 129.1, 127.1, 79.7, 52.0, 44.3, 28.3 ppm.

1.11 Synthesis of *tert*-butyl(4-(hydroxymethyl)benzyl)carbamate (9)

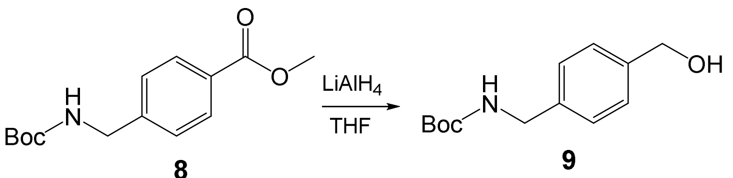

8 **9**

To a solution of **8** (224 mg, 0.84 mmol), in THF, $LiAlH_4$ (160 mg, 4.2 mmol,) was added at 0°C under inert atmospere. The resulting mixture was stirred overnight at room temperature. Then, it was diluted with brine and NaOH 8 M (10 mL), and extracted with ethyl acetate. The organic layer was dried over anhydrous Na_2SO_4, filtrated and concentrated under reduced pressure to obtain the pure product (160 mg, 0.67 mmol) in 80% yield. The spectroscopic data match those reported in the literature.[4]

^{1}H NMR (400 MHz, $CDCl_3$): δ 1.43 (s, 9H, *t*-Bu), 4.26 (d, J = 6.1 Hz, 2H, CH_2), 4.64 (s, 2H, CH_2), 4.86 (s, 1H, NH), 7.21-7.31 (m, 4H, Ar) ppm.

^{13}C NMR (100 MHz, $CDCl_3$): δ 28.4, 44.4, 64.9, 79.5, 127.2, 127.6, 138.3, 140.0, 155.9 ppm.

1.12 Synthesis of *tert*-butyl (4-((prop-2-yn-1-yloxy)methyl)benzyl)carbamate (10)

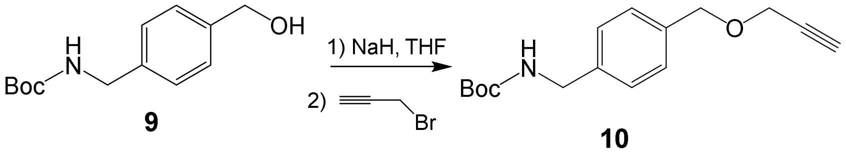

9 **10**

To a solution of **9** (160 mg, 0.67 mmol) and NaH 60% (32 mg, 0.81 mmol) in THF, propargyl bromide (120 mg, 0.81 mmol) was added dropwise at 0°C under inert atmosphere. The resulting mixture was stirred overnight at room temperature. Then, it was diluted with distilled water, and extracted wit ethyl acetate. The organic layer was dried over anhydrous Na_2SO_4, filtrated and concentrated under reduced pressure to obtain the crude that was chromatographed over a silica gel column eluting with cyclohexane/ethylacetate 1:1. The pure product was obtained as a pale yellow oil (92 mg, 0.33 mmol) in 50% yield. Yield was not optimized.

^{1}H NMR (400 MHz, $CDCl_3$): δ 1.46 (s, 9H, *t*-Bu), 2.46 (s, 1H, CH), 4.17 (s, 2H, CH_2), 4.32 (m, 2H, CH_2), 4.59 (s, 2H, CH_2), 4.81 (s, 1H, NH), 7.24-7.35 (m, 4H, Ar) ppm.
^{13}C NMR (100 MHz, $CDCl_3$): δ 28.4, 44.4, 57.0, 65.0, 71.2, 74.6, 127.2, 128.4, 136.3, 140.0, 155.9 ppm.

1.13 Synthesis of (4-((prop-2-yn-1-yloxy)methyl)phenyl)methanamine (11)

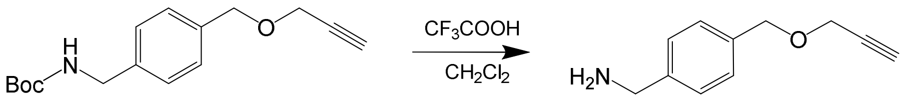

10 **11**

To a solution of **10** (92 mg, 0.33 mmol) in CH_2Cl_2 (5 mL), trifluoroacetic acid (0.76 mL, 9.9 mmol) was added dropwise under magnetic stirring, and the solution was reacted for 2 h. Then the mixture was neutralized with $NaHCO_3$ until pH 8-9. After extraction with CH_2Cl_2, drying over Na_2SO_4, and evaporation under vacuo the product **11** was obtained in 80% yield as a dark yellow oil.

^{1}H NMR (400 MHz, $CDCl_3$): δ 2.46 (t, J = 2.5 Hz, 1H, CH), 3.86 (s, 2H, CH_2), 4.16 (d, J = 2.5 Hz, 2H, CH_2), 4.59 (s, 2H, CH_2), 7.27-7.34 (m, 4H, Ar) ppm.

^{13}C NMR (100 MHz, $CDCl_3$): δ 46.1, 57.0, 71.2, 74.6, 79.6, 127.2, 128.4, 135.8, 142.7 ppm.

1.14 Synthesis of alkyne 13

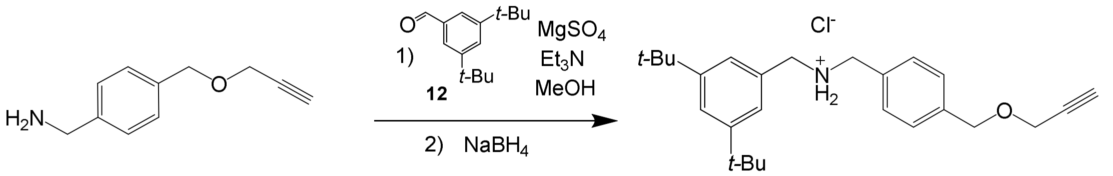

MgSO$_4$ was added to a solution of **11** (180 mg, 1.02 mmol) in MeOH (10 mL) followed by 3,5-di-*tert*-butylbenzaldehyde **12** (220 mg, 1.02 mmol) and Et_3N (1µL), and the mixture was refluxed under stirring for 4 hs. Afterwards $NaBH_4$ (190 mg, 5.1 mmol) was added continuing refluxing overnight. The cooled reaction mixture was then treated with 2H HCl until pH=2, and a precipitate was formed. After evaporation of the solvent the residue was suspended in NaOH 8N, extracted with CH_2Cl_2, dried over Na_2SO_4 and evaporated under vacuo. The crude product was then dissolved in ethyl ether and treated with gaseous HCl obtained by dropping concentrated H_2SO_4 onto NaCl. Evaporation of the solvent affords the desired compound in 80% yield.

^{1}H NMR (400 MHz, $CDCl_3$) δ 1.32 (s, 18H, *t*-Bu), 2.45 (t, J =2.4 Hz, 1H, CH), 3.83 (t, J=4.5 Hz, 2H, CH_2), 3.89 (t, J=4.5 Hz, 2H, CH_2), 4.11 (d, J=2.4 Hz, 2H, CH_2), 4.51 (s, 2H, CH_2), 7.32 (d, J=1.8 Hz, 2H, Ar), 7.33 (d, J=8 Hz, 2H, Ar), 7.40 (t, J=1.8 Hz, 1H, Ar), 7.48 (d, J =8 Hz, 2H, Ar), 10.29 (s, 2H, NH_2^+) ppm.

^{13}C NMR (100 MHz, $CDCl_3$) δ 31.4, 35.0, 47.9, 49.0, 57.3, 70.9, 74.8, 79.3, 123.1, 124.6, 128.4, 130.6, 138.6, 138.8, 151.8 ppm.

1.15 Synthesis of 1-(azidomethyl)-3,5-di-*tert*-butylbenzene 14[5]

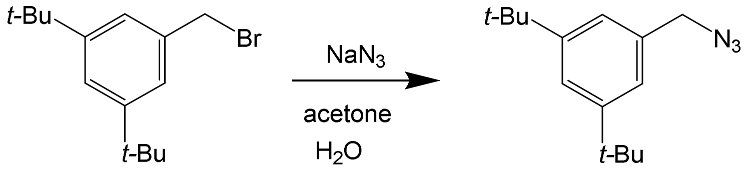

14

The reaction was carried out following the procedure of Feringa et al.[6] Azido compounds are known for their explosive behavior, and must be handled with the necessary care.[7]

To a stirred solution of the bromide (1.0 eq) in a 50 mL water/acetone mixture (1:4) was added NaN_3 (1.5 eq). The resulting suspension was stirred at room temperature for 24 hours. DCM was added to the mixture and the organic layer was separated. The aqueous layer was extracted with 3 x 10 mL aliquots of DCM and the combined organic layers were dried over Na_2SO_4. Solvent was removed under reduced pressure, and the azide **14** was sufficiently pure to use without further work up.

^{1}H NMR (400 MHz, $CDCl_3$) δ 1.34 (s, 18H, *t*-Bu), 4.34 (s, 2H, CH_2), 7.14 (d, J = 1.9 Hz, 2H, Ar), 7.41 (t, J=1.9 Hz, 1H, Ar) ppm.

^{13}C NMR (100 MHz, $CDCl_3$) δ 31.4, 34.8, 55.5, 122.3, 122.4,134.5, 151.4 ppm.

1.16 Synthesis of dumbbell D

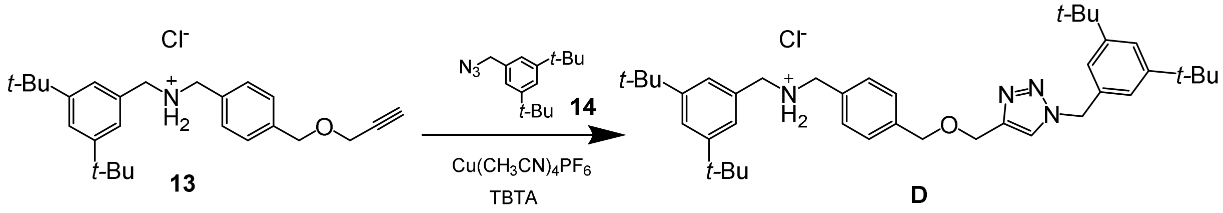

To a stirred solution of **13** (273 mg, 0.66 mmol) in CH_2Cl_2 (30 mL) a solution of **14** (321 mg, 1.31 mmol) in CH_2Cl_2 (15 mL), $Cu(CH_3CN)_4PF_6$ (245 mg, 0.66 mmol) and tris(benzyltriazolylmethyl)amine (TBTA) in catalytic amounts were added under nitrogen, and the resulting mixture was stirred at room temperature overnight. Afterwards the mixture was washed with EDTA and brine and then extracted with CH_2Cl_2. The organic layers were collected, dried (Na_2SO_4), concentrated, and the resulting crude was purified by a silica gel column using CH_2Cl_2 until CH_2Cl_2/MeOH 9/1. Dumbbell **D** (130 mg, 0.198 mmol) is obtained in 30% not optimized yield.

^{1}H NMR (400 MHz, $CDCl_3$): δ 1.29 (s, 18H, *t*-Bu), 1.32 (s, 18H, *t*-Bu), 3.78 (s, 2H, CH_2), 3.82 (s, 2H, CH_2), 4.56 (s, 2H, CH_2), 4.66 (s, 2H, CH_2), 5.49 (s, 2H, CH_2) 7.11 (s, 2H, Ar), 7.16 (s, 2H, Ar), 7.26-7.34 (m, 5H, Ar,), 7.41 (s, 1H, Ar), 7.46 (s, 1H, Trz) ppm.
^{13}C NMR (100 MHz, $CDCl_3$): δ 31.3, 31.5, 34.8, 34.9, 54.8, 63.7, 72.2, 72.2, 77.2, 121.0, 122.3, 122.4, 122.8, 128.1, 128.3, 133.6, 136.5, 145.4, 150.8, 151.8 ppm.
HRMS (ESI) *m/z*: $[M]^+$ calcd. for $C_{41}H_{59}N_4O^+$: 623.4683; found 623.4664.

1.17 Synthesis of Rotaxane (Rot2+)

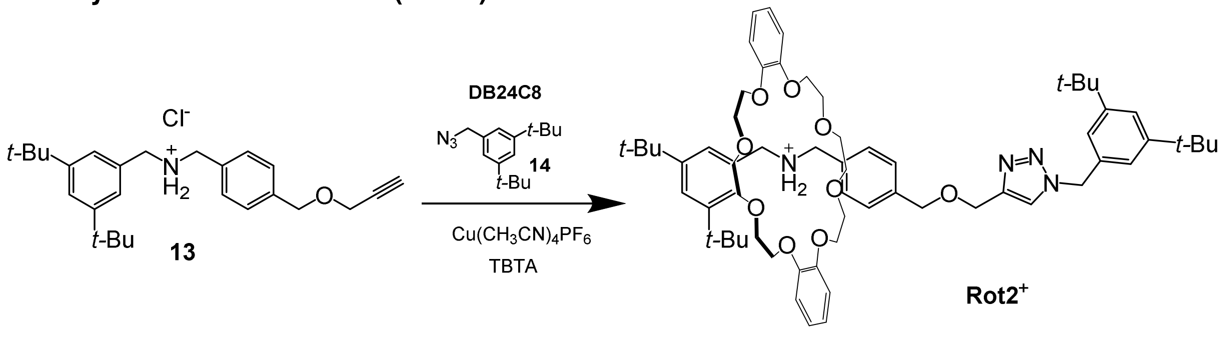

A solution of half thread **13** (273 mg, 0.66 mmol) and **DB24C8** (588 mg, 1.31 mmol) in CH_2Cl_2 (35 mL) was stirred under nitrogen. After 2 hours, a solution of **14** (321 mg, 1.31 mmol) in CH_2Cl_2 (15 mL), $Cu(CH_3CN)_4PF_6$ (245 mg, 0.66 mmol) and TBTA in catalytic amounts were added, and the resulting mixture was stirred at room temperature overnight. Afterwards the crude was washed with EDTA and then extracted with CH_2Cl_2. The organic layers were collected, dried (Na_2SO_4), concentrated, and the resulting crude was purified by a silica gel column using CH_2Cl_2 until CH_2Cl_2/MeOH 9/1. Rotaxane **Rot2**$^+$ (276 mg, 0.25 mmol) is obtained as a brown oil in 37% not optimized yield.

^{1}H NMR (400 MHz, $CDCl_3$) δ 1.19 (s, 18H, *t*-Bu), 1.28 (s, 18H, *t*-Bu), 3.43-3.50 (m, 4H, CH_2), 3.58-3.64 (m, 4H, CH_2), 3.69-3.76 (m, 4H, CH_2), 3.79-3.85 (m, 4H, CH_2), 3.89-3.93 (m, 2H, CH_2), 4.00-4.16 (m, 6H, CH_2), 4.39 (s, 2H, CH_2), 4.54 (s, 2H, CH_2), 4.53-4.59 (m, 2H, CH_2), 4.65-4.72 (m, 2H, CH_2), 5.52 (s, 2H, CH_2), 6.74-6.79 (m, 4H, Ar), 6.81-6.86 (m, 4H Ar), 7.02 (d, J = 8.4 Hz, 2H, Ar), 7.14 (d, J = 8.4 Hz, 2H, Ar), 7.14 (d, J = 1.8 Hz, 2H, Ar),7.27 (d, J = 1.8 Hz, 2H, Ar), 7.36 (t, J = 1.8 Hz, 1H, Ar), 7.39 (t, J = 1.8 Hz, 1H, Ar), 7.62 (s, 1H, Trz), 7.64 (*br*s, 2H, NH_2^+) ppm.

^{13}C NMR (100 MHz, $CDCl_3$) δ 31.2,31.3, 34.7, 34.8, 47.2, 52.5, 53.0, 54.0, 54.7, 63.4, 67.9, 70.1, 70.5, 71.2, 112.5, 121.6, 121.7, 122.5, 122.6, 123.0, 123.2, 123.6, 123.7, 127.6, 128.0, 128.5, 129.0, 129.2, 130.3, 131.2, 133.9, 134.8, 139.2, 144.3, 144.9, 147.3, 151.4, 151.6 ppm.

HRMS (ESI) *m/z*: [M]$^+$ calcd. for $C_{65}H_{91}N_4O_9^+$: 1071.6781; found 1071.6839

1.18 Synthesis of Rotaxane (Rot1+•)

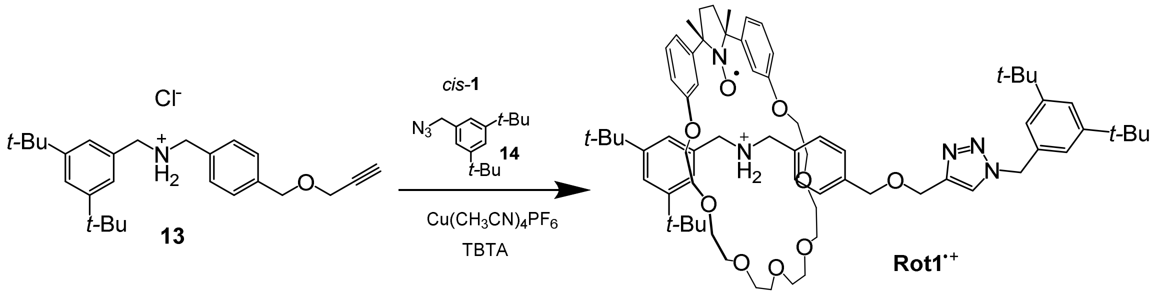

Alkyne **13** (38 mg, 0.1 mmol) and *cis*-**1** (53 mg, 0.097 mmol) were dissolved in CH_2Cl_2 (4.8 mL) under nitrogen. After 2 hours, a solution of azide **14** (49 mg, 0.2 mmol), $Cu(CH_3CN)_4PF_6$ (37 mg, 0.1 mmol)

and TBTA in catalytic amounts in CH_2Cl_2 (2 mL) were added, and the resulting mixture was stirred at room temperature overnight. Afterwards the mixture was washed with EDTA and then extracted with CH_2Cl_2. The organic layers were collected, dried (Na_2SO_4), concentrated, and the resulting crude was purified by a silica gel column starting CH_2Cl_2 until CH_2Cl_2/MeOH 9/1. **Rot1**$^{+•}$ (0.020 g, 0.017 mmol) is obtained as an orange oil in 17% not optimized yield.
EPR (CH_2Cl_2): a_N= 14.39 G, g=2.0061.

^{1}H NMR (400 MHz, $CDCl_3$): d 1.30, 1.33 (s, *t*-Bu), 3.53-3.96 (m, CH_2), 4.12-4.24 (m, CH_2), 4.56 (s, CH_2), 4.66 (s, CH_2), 5.50 (s, CH_2), 7.08-7.47 (m, Ar) ppm.

HRMS (ESI) *m/z*: $[M+H]^{2+}$ calcd. for $C_{71}H_{101}N_5O_9^{•+}$ 584,3836; found 584.3831.

1.19 GC-MS measurements.

The GC-MS analysis were carried using Network GC-system 6890N (Agilent technologies) with a time delay of 4 minutes, flow rate of 1.5 ml/s, starting from an initial temperature of 50 °C up to a temperature of 270 °C, through a speed ramp of 30 °C/min. A solution of 4-methoxybenzyl alcohol (0.01 M) and radical TEMPO or *cis*-**1**$^•$ (0.001 M) in CH_3CN was prepared and fluxed with oxygen. Then, the necessary aliquots of CAN (0.002 M or 0.004 M) were added, and the mixtures refluxed while stirring. Then the reactions were followed by GC-MS analysis at regular time intervals. In the reactions conducted under inert atmosphere the mixtures were fluxed under nitrogen.
The conversions were calculated by comparing the integrals of the chromatographic peaks of the alcohol and the aldehyde in the presence of an internal standard (1,2,4-trichlorobenzene, 0.003 M).

1.20 Electrochemical Measurements.

Cyclic voltammetric (CV) experiments were carried out in argon-purged CH_2Cl_2 (Supplementary Figure 29) and CH_3CN with an Autolab 30 multipurpose instrument interfaced to a PC. The working electrode was a glassy carbon electrode (Amel, 0.07 cm^2), carefully polished with an alumina-water slurry on a felt surface, immediately before use. The counter electrode was a Pt wire, separated from the solution by a frit, an Ag wire was employed as a quasi-reference electrode and decamethylferrocene was present as an internal standard ($E_{1/2}$= -0.086 V vs SCE in CH_2Cl_2; $E_{1/2}$= -0.11 V vs SCE in CH_3CN). The concentration of the examined compounds was ranging from 0.1 to 0.5 mM. Tetrabutylammonium hexafluorophosphate ($TBAPF_6$) and tetraethylammonium hexafluorophosphate ($TEAPF_6$) were added as supporting electrolyte in CH_2Cl_2 and CH_3CN respectively, in a 100-fold proportion with respect to the sample concentration. Cyclic voltammograms were obtained at scan rates varying from 50 to 2000 mVs^{-1}. The IR compensation was used and every effort was made throughout the experiments in order to minimize the resistance of the solution. The electrochemical reversibility of the voltammetric wave of ferrocene was taken as an indicator of the absence of uncompensated resistance effects. For reversible processes the

halfwave potential values were obtained from the average of the cathodic and anodic cyclic voltammetric waves.

1.21 ESR Measurements of Rot1$^{+\bullet}$ in the presence of a base.

When one equivalent of the strong base phosphazene P1-*t*-Bu, which is strong enough to deprotonate the NH_2^+ centre, is added to a CH_3CN solution of **Rot1**$^{\bullet+}$, a change in the EPR nitrogen coupling is observed (see Supplementary Figure 30, red line). Specifically, a_N value increases from 14.39 G to 14.63 G. The subsequent addition of a stoichiometric amount of trifluoroacetic acid (TFA) to the rotaxane, after the base addition, results in the quantitative recovery of the initial EPR spectrum. This observation is consistent with the system reverting to its state where the macrocycle predominantly encircles the ammonium station.

2. Supplementary Figures

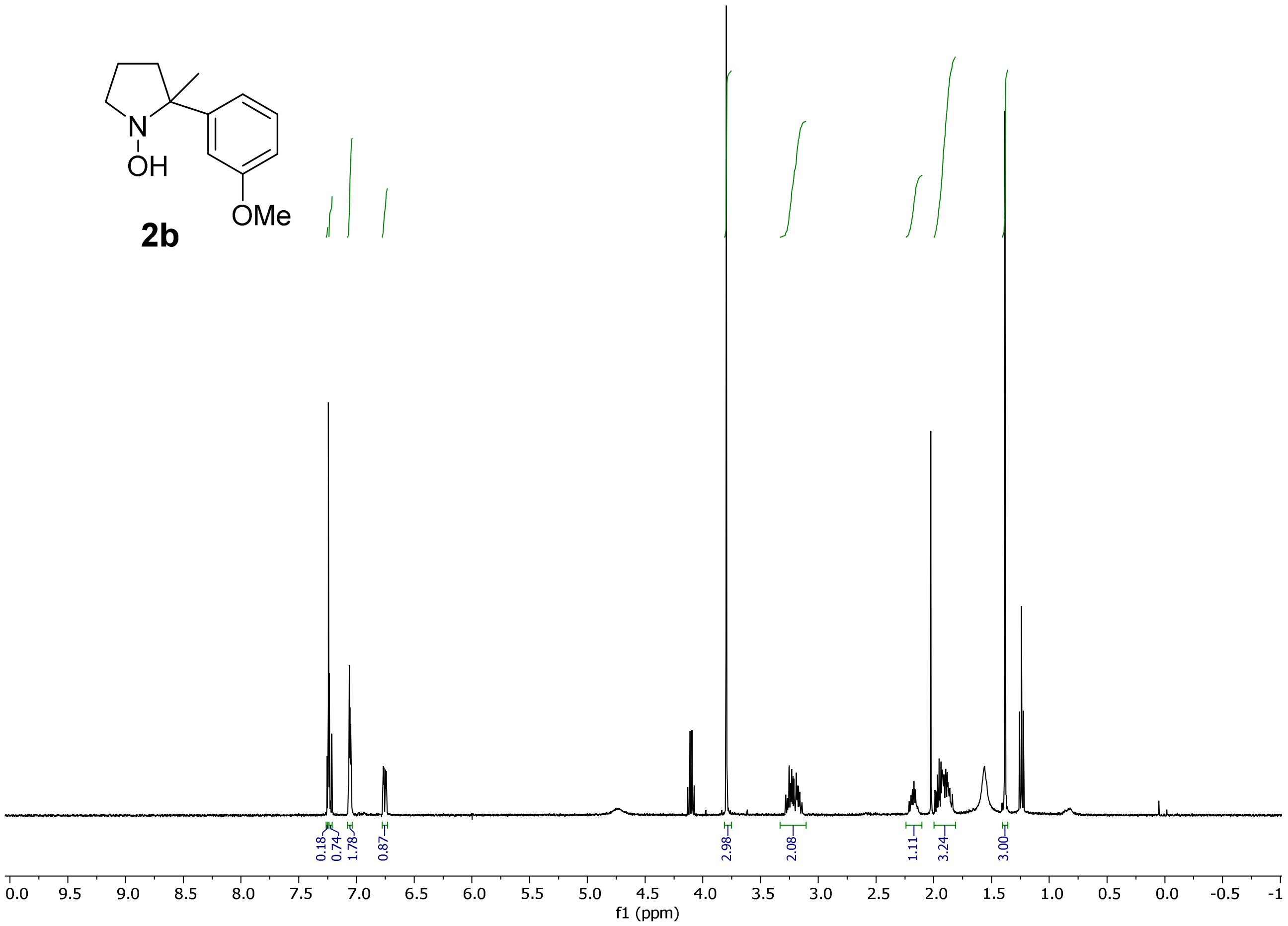

Supplementary Figure 1. ^{1}H NMR spectrum (400 MHz, $CDCl_3$) of **2b**.

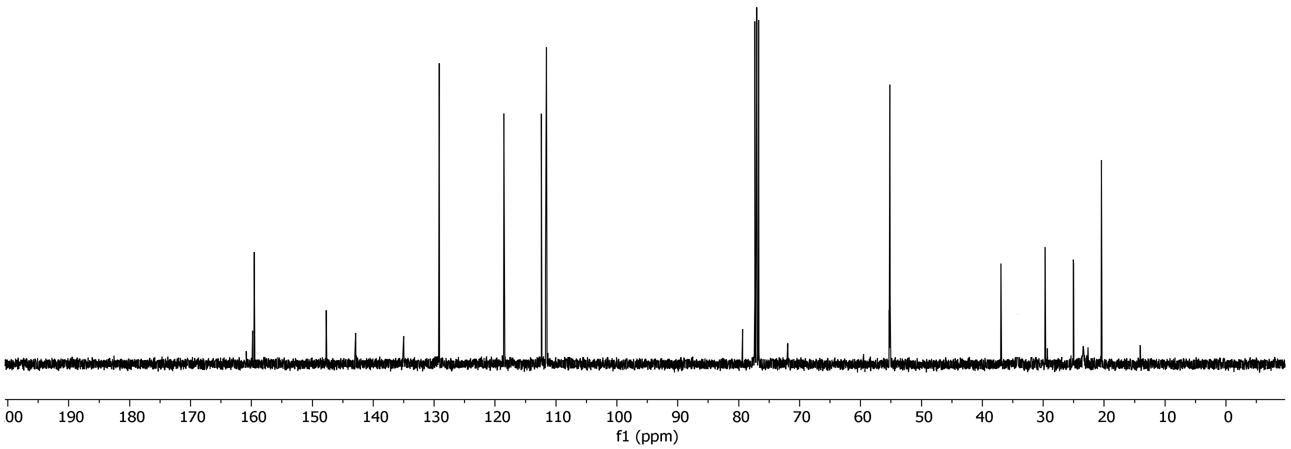

Supplementary Figure 2. ^{13}C NMR spectrum (100 MHz, $CDCl_3$) of **2b**.

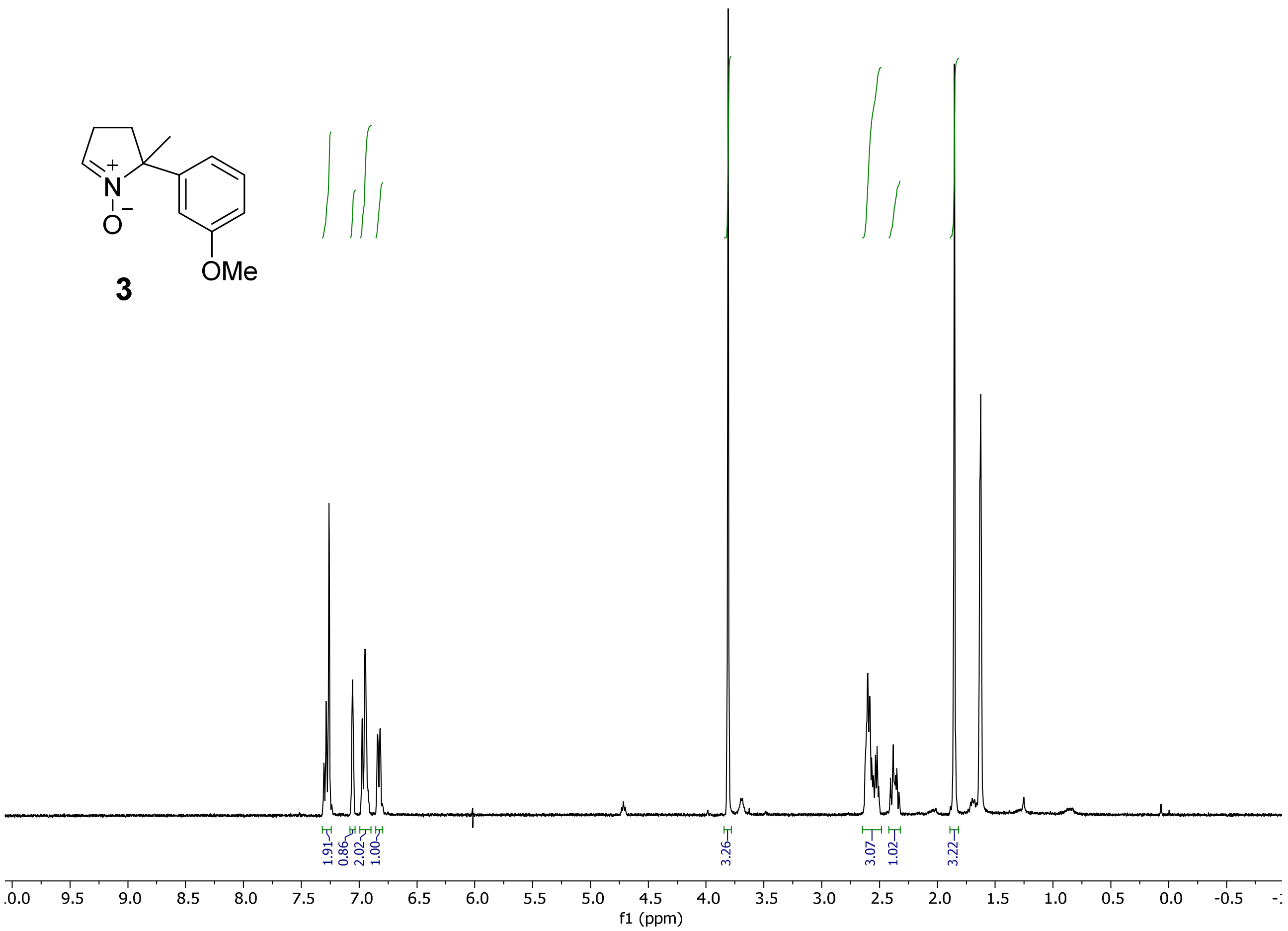

Supplementary Figure 3. ^{1}H NMR spectrum (400 MHz, $CDCl_3$) of **3**.

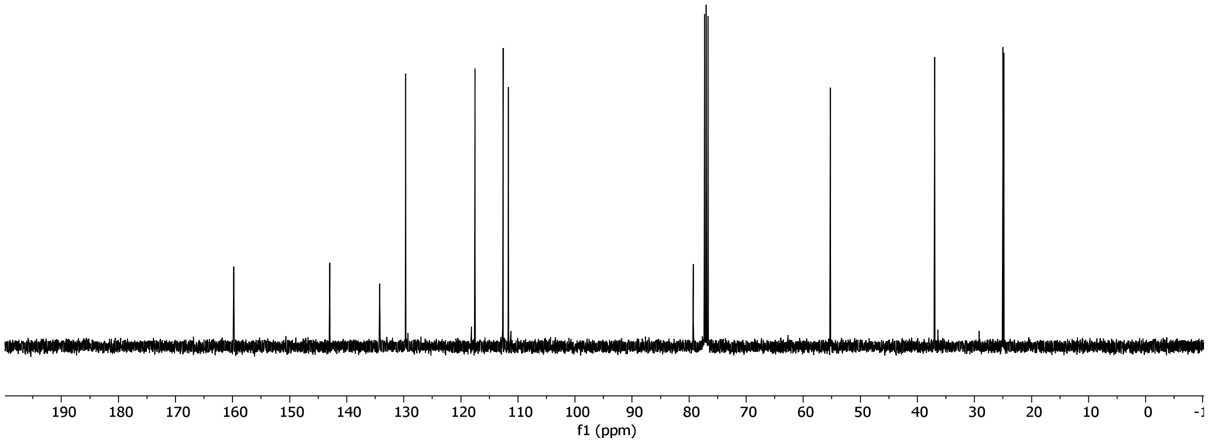

Supplementary Figure 4. ^{13}C NMR spectrum (100 MHz, $CDCl_3$) of **3**.

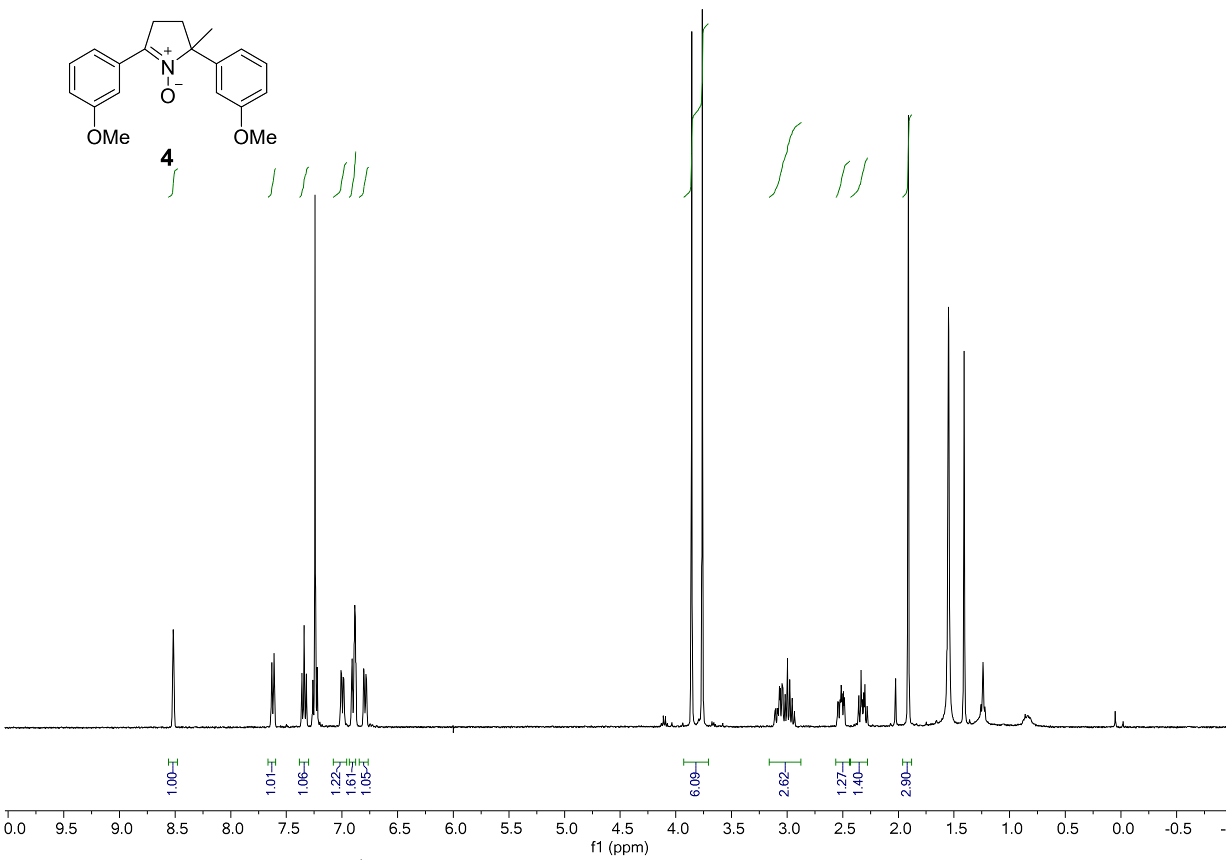

Supplementary Figure 5. ^{1}H NMR spectrum (400 MHz, $CDCl_3$) of **4**.

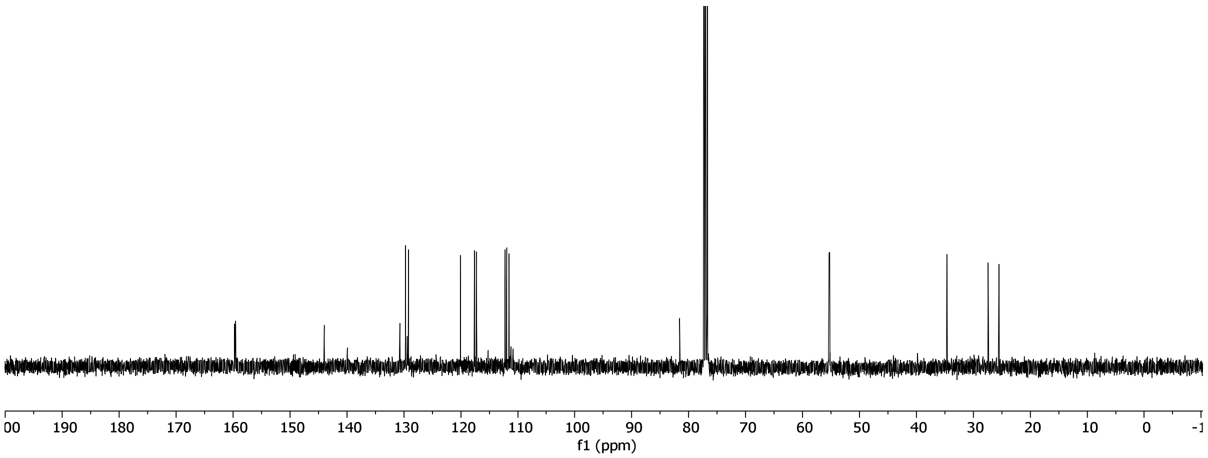

Supplementary Figure 6. ^{13}C NMR spectrum (100 MHz, $CDCl_3$) of **4**.

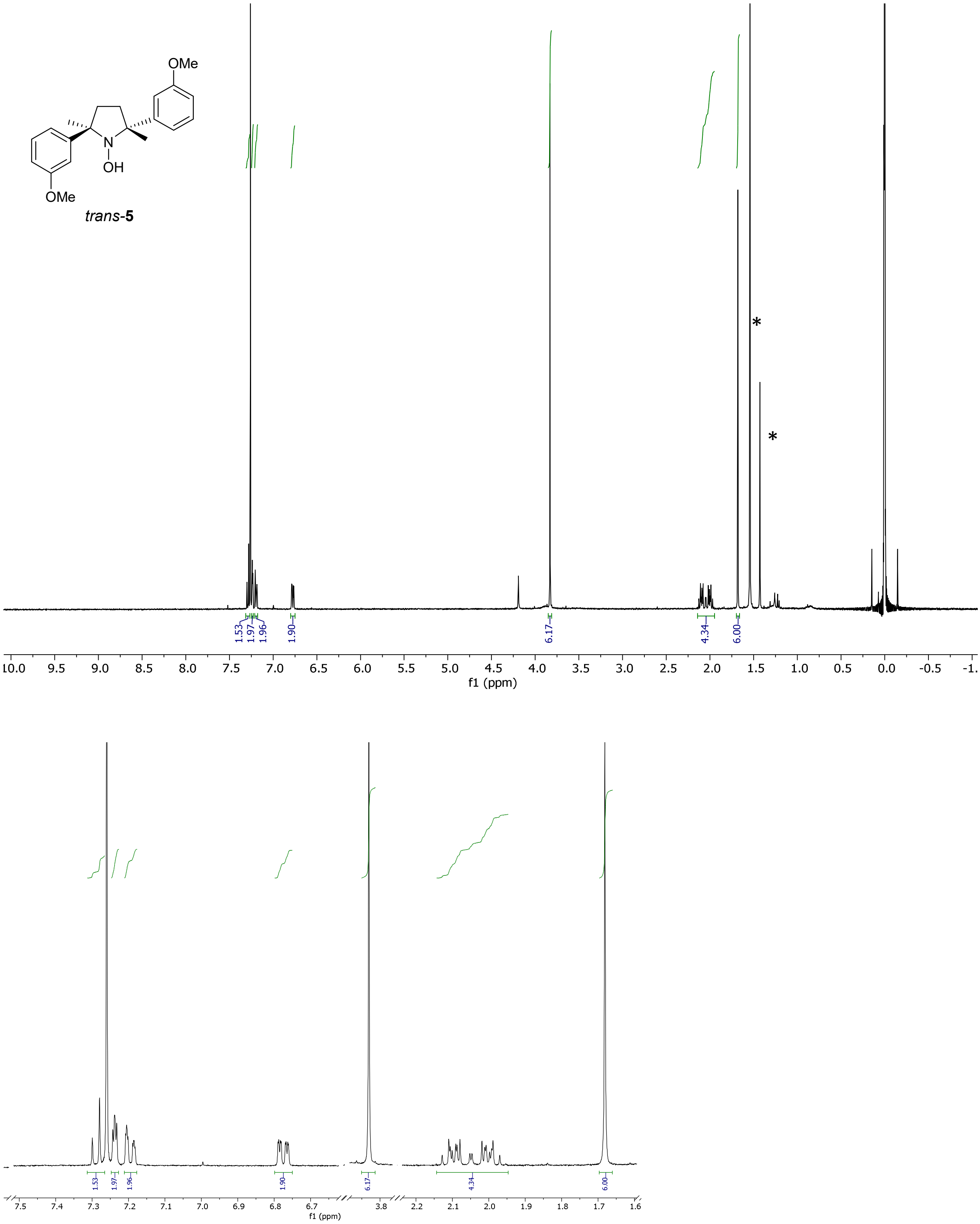

Supplementary Figure 7. ^{1}H NMR spectrum (400 MHz, $CDCl_3$) of *trans*-**5** (up); and selected spectral regions (down). Stars indicate residual solvents.

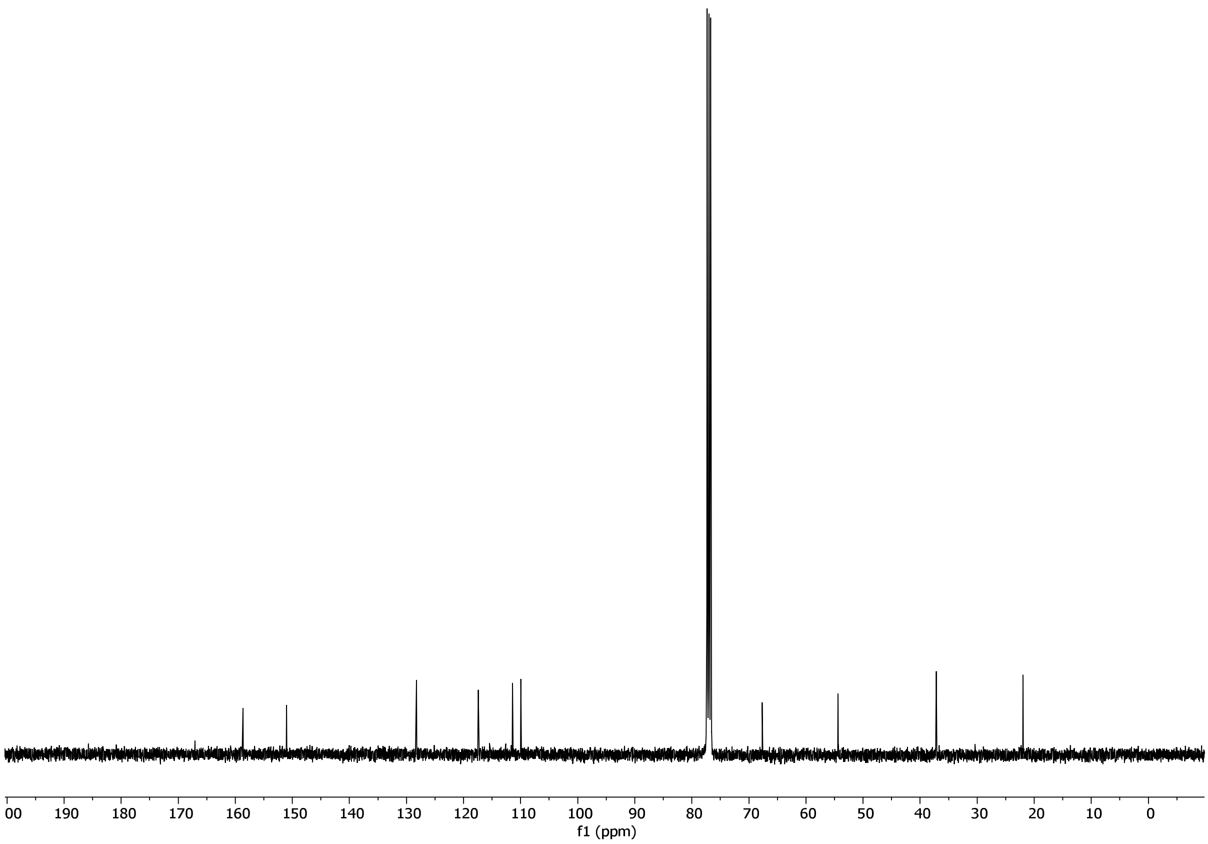

Supplementary Figure 8. ^{13}C NMR spectrum (100 MHz, $CDCl_3$) of *trans*-**5**.

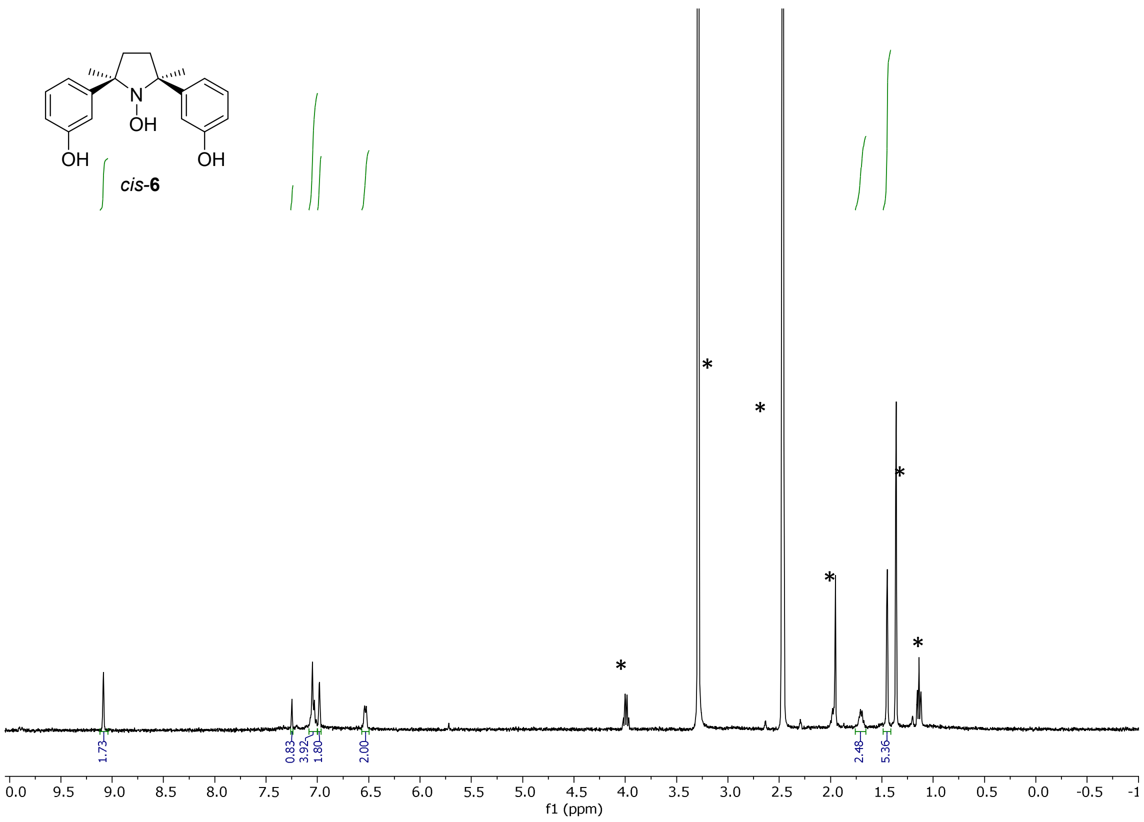

Supplementary Figure 9. ^{1}H NMR spectrum (400 MHz, d^6-DMSO) of *cis*-**6**. Stars indicate residual solvents.

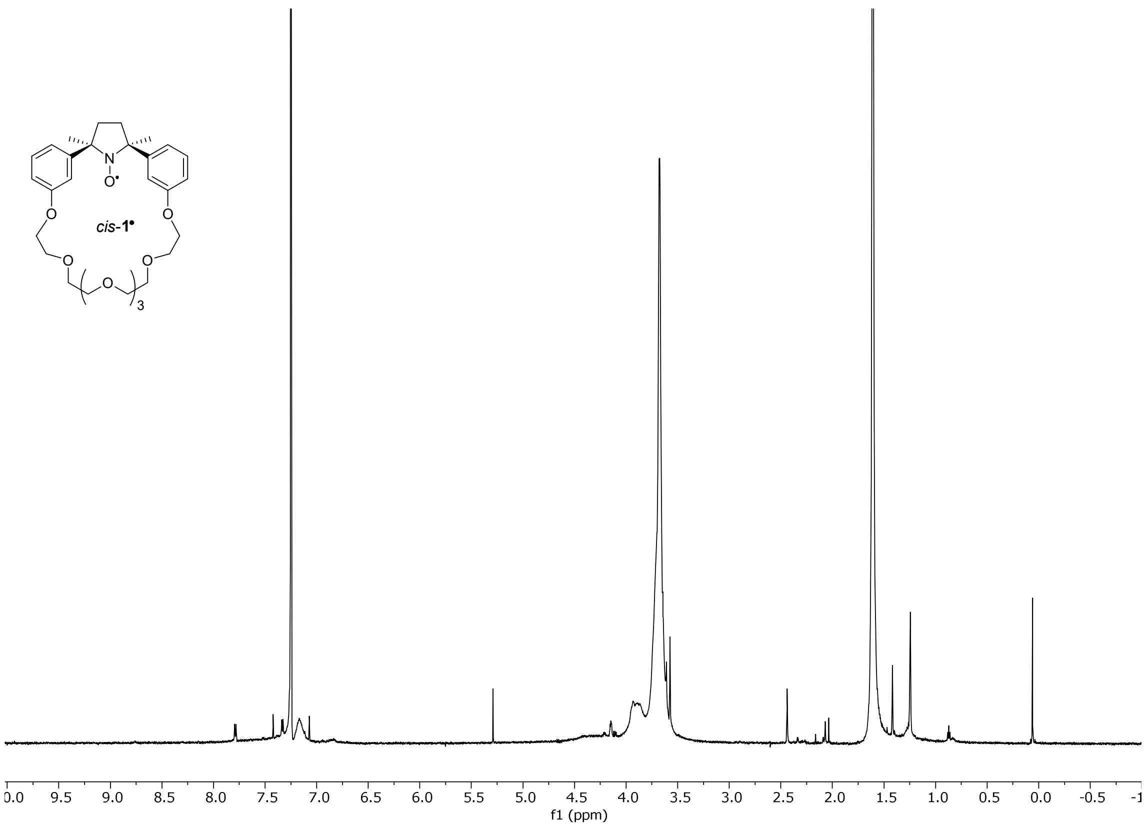

Supplementary Figure 10. ^{1}H NMR spectrum (400 MHz, $CDCl_3$) of *cis*-**1**$^{\bullet}$.

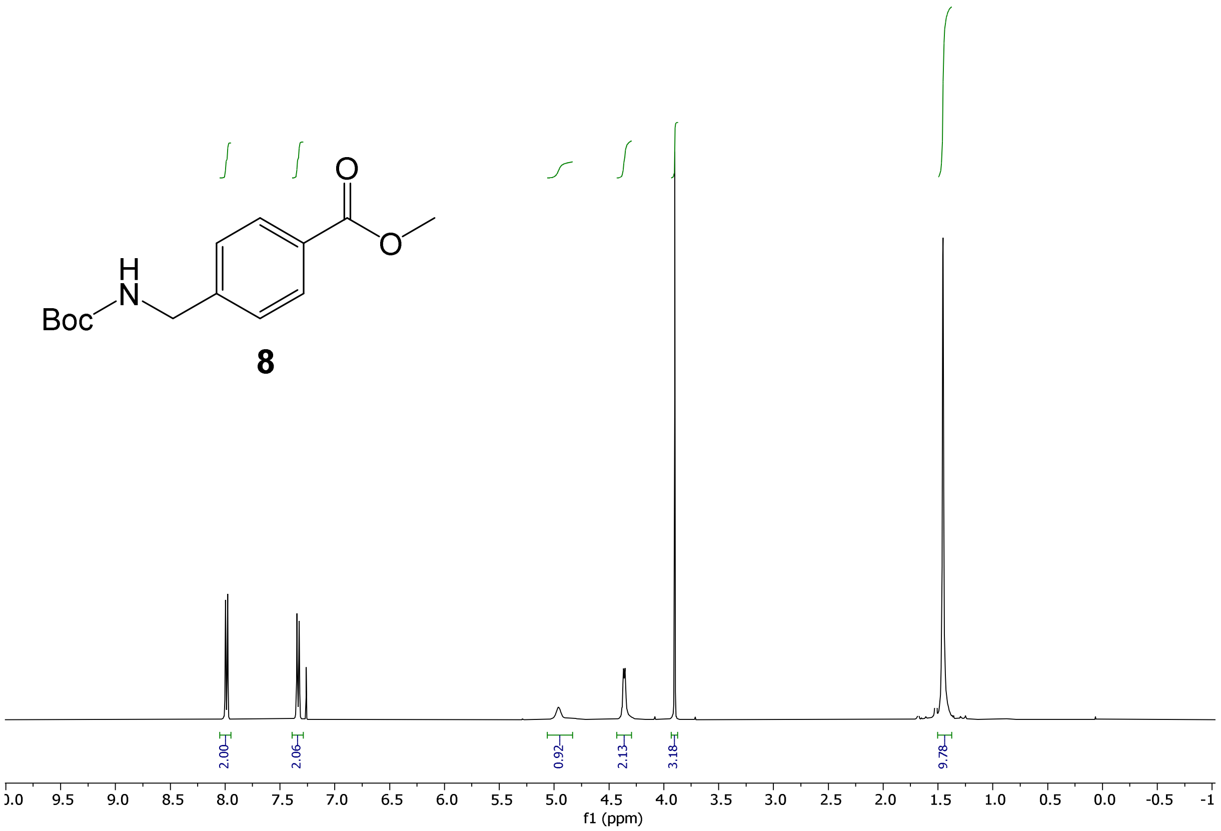

Supplementary Figure 11. ^{1}H NMR spectrum (400 MHz, $CDCl_3$) of **8**.

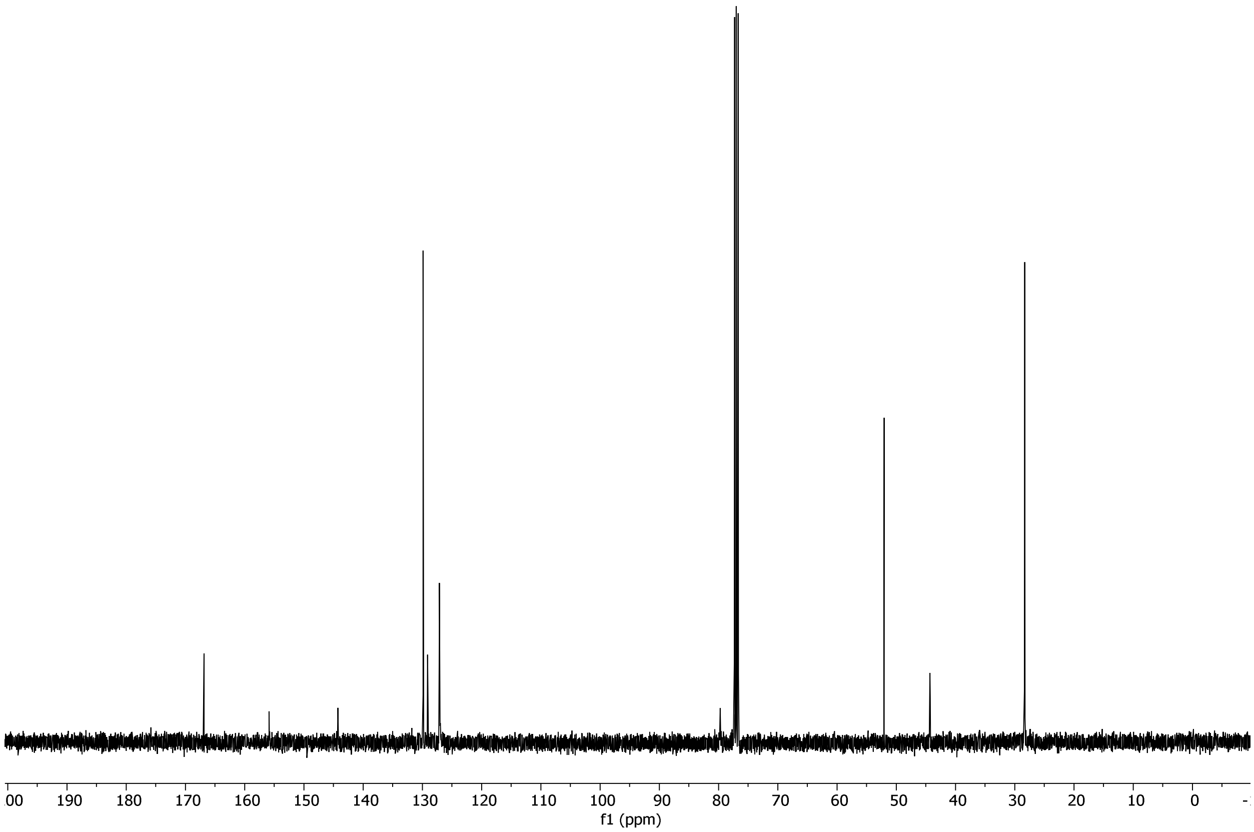

Supplementary Figure 12. ^{13}C NMR spectrum (100 MHz, $CDCl_3$) of **8**.

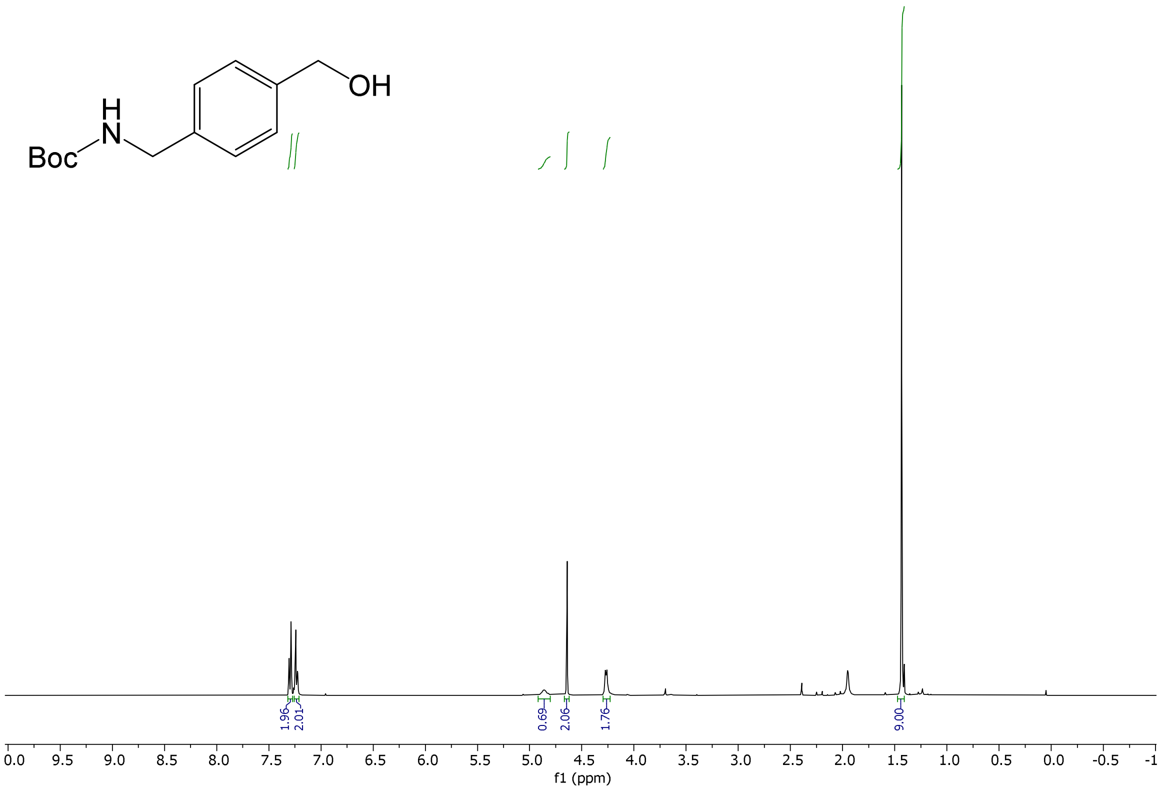

Supplementary Figure 13. ^{1}H NMR spectrum (400 MHz, $CDCl_3$) of **9**.

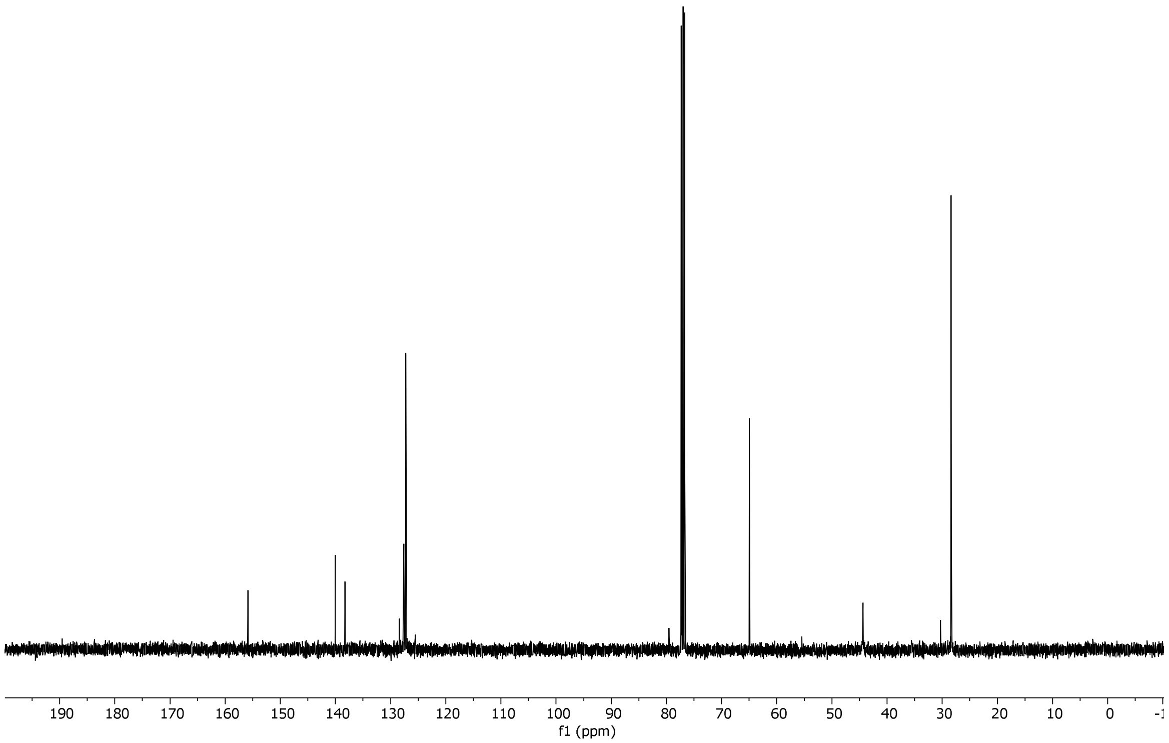

Supplementary Figure 14. ^{13}C NMR spectrum (100 MHz, $CDCl_3$) of **9**.

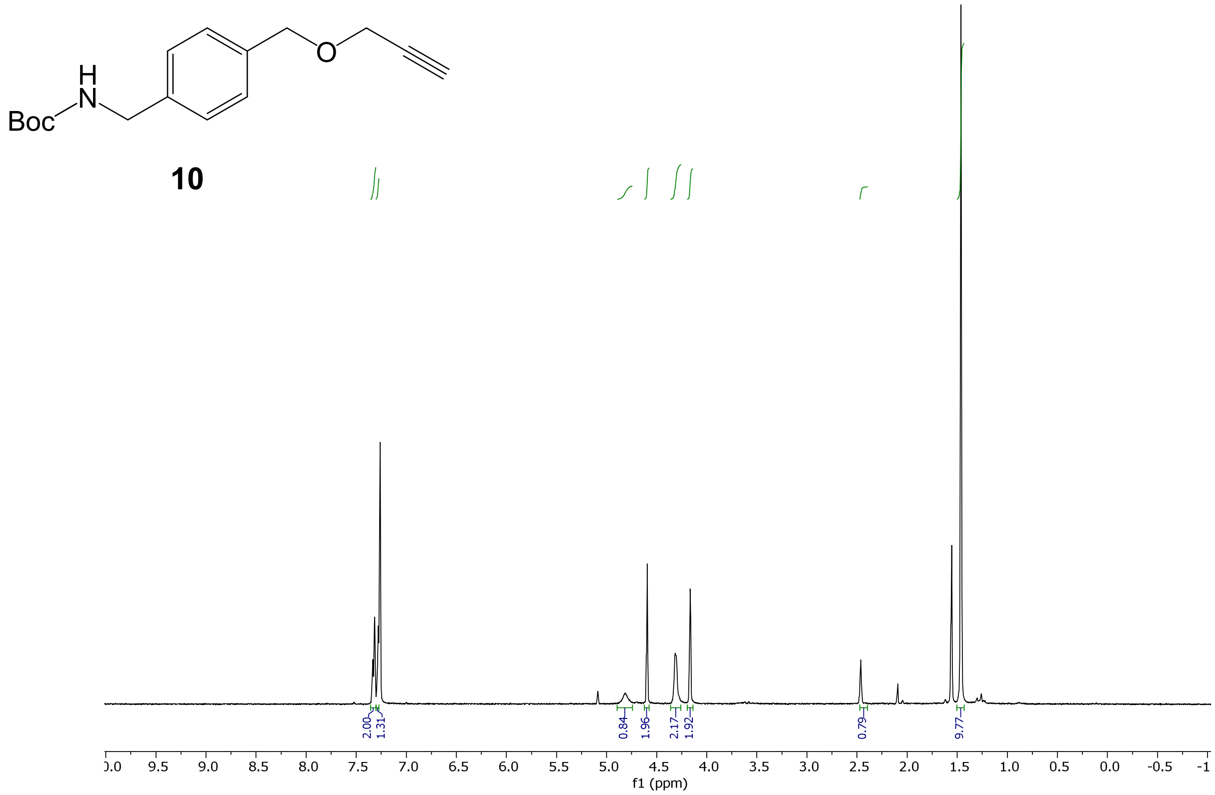

Supplementary Figure 15. ^{1}H NMR spectrum (400 MHz, $CDCl_3$) of **10**.

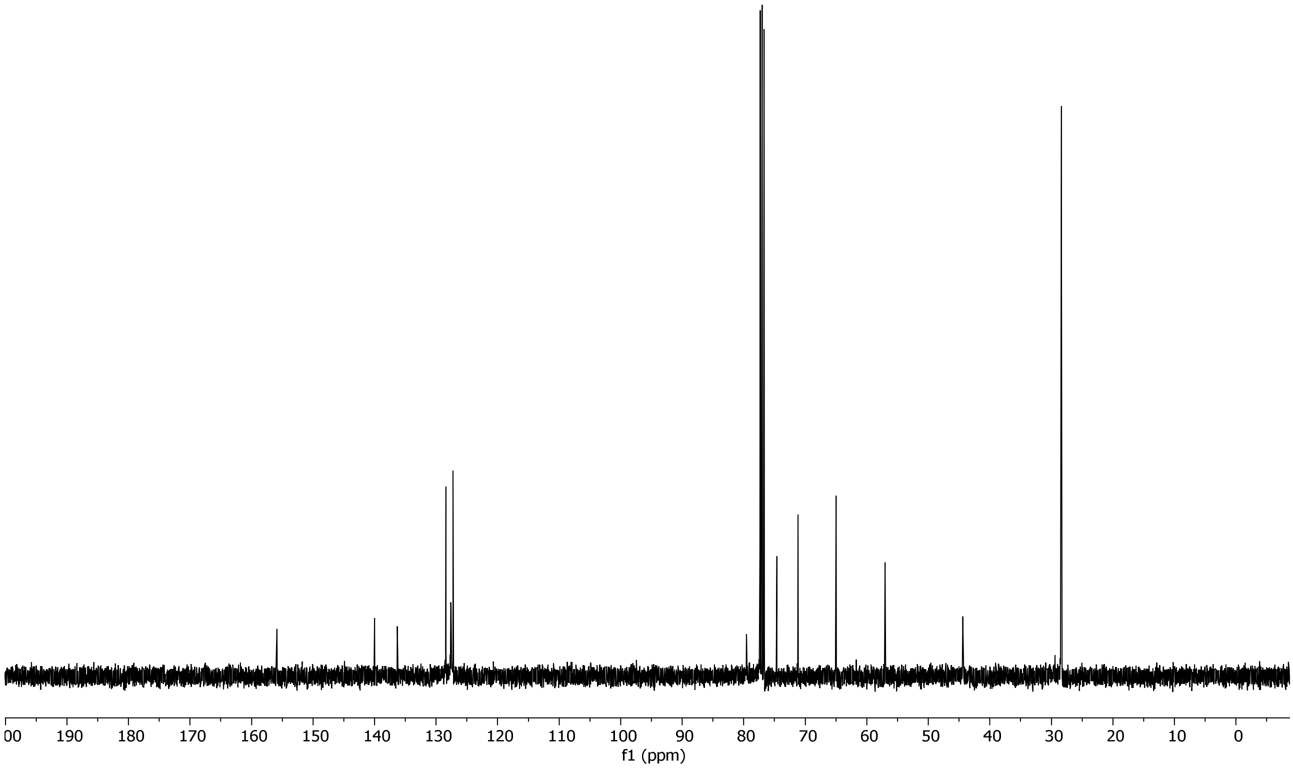

Supplementary Figure 16. ^{13}C NMR spectrum (100 MHz, $CDCl_3$) of **10**.

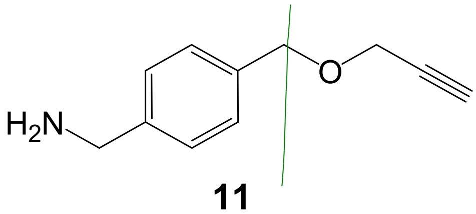

11

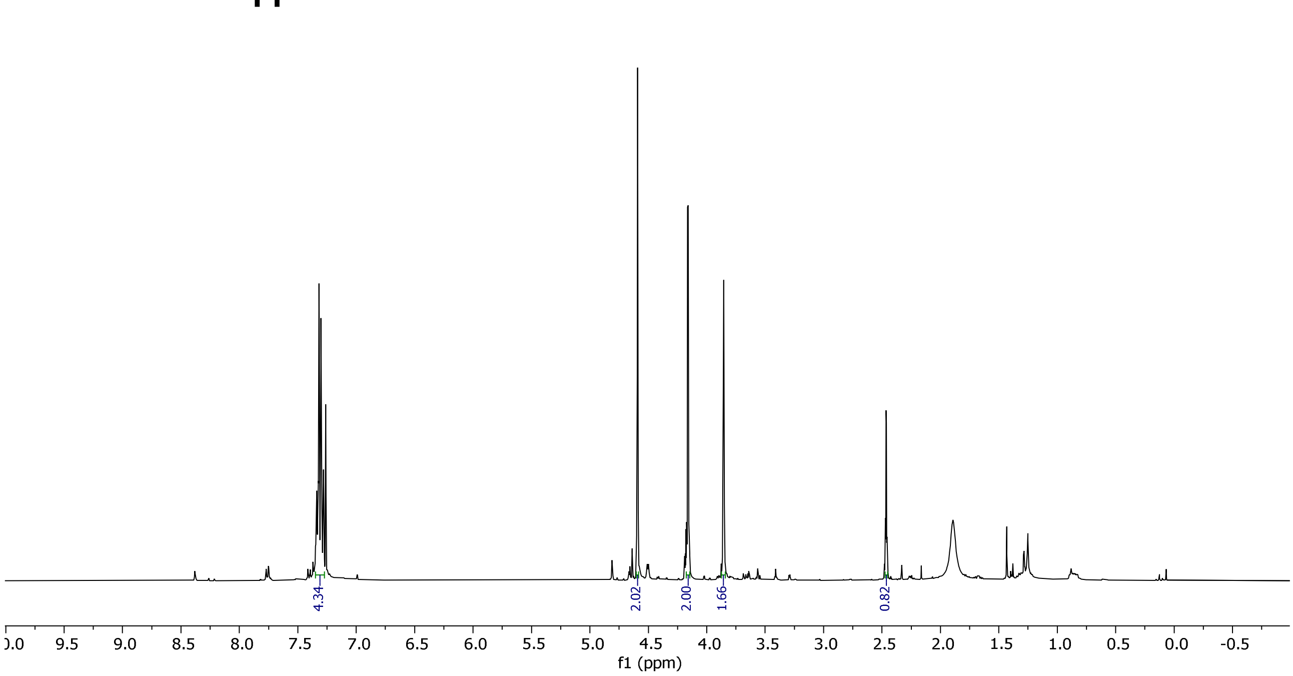

Supplementary Figure 17. ^{1}H NMR spectrum (400 MHz, $CDCl_3$) of **11**.

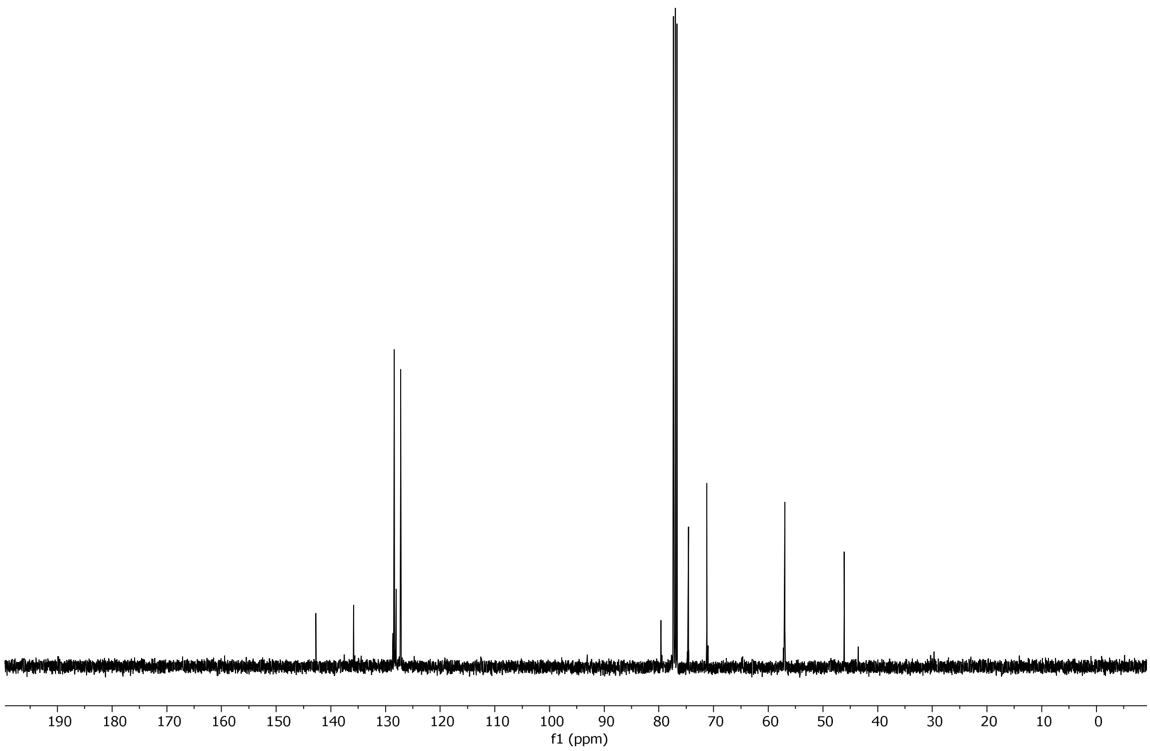

Supplementary Figure 18. ^{13}C NMR spectrum (100 MHz, $CDCl_3$) of **11**.

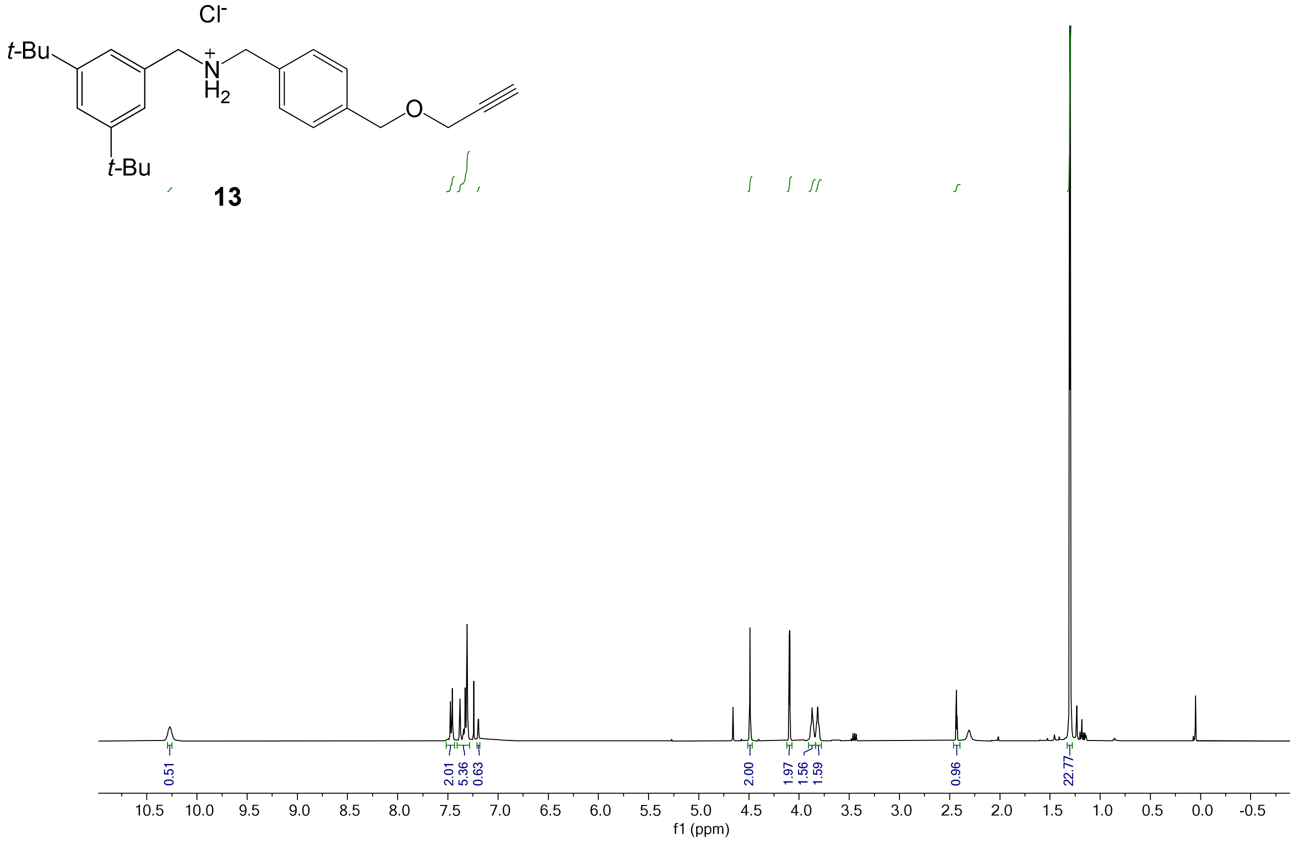

Supplementary Figure 19. ^{1}H NMR spectrum (400 MHz, $CDCl_3$) of **13**.

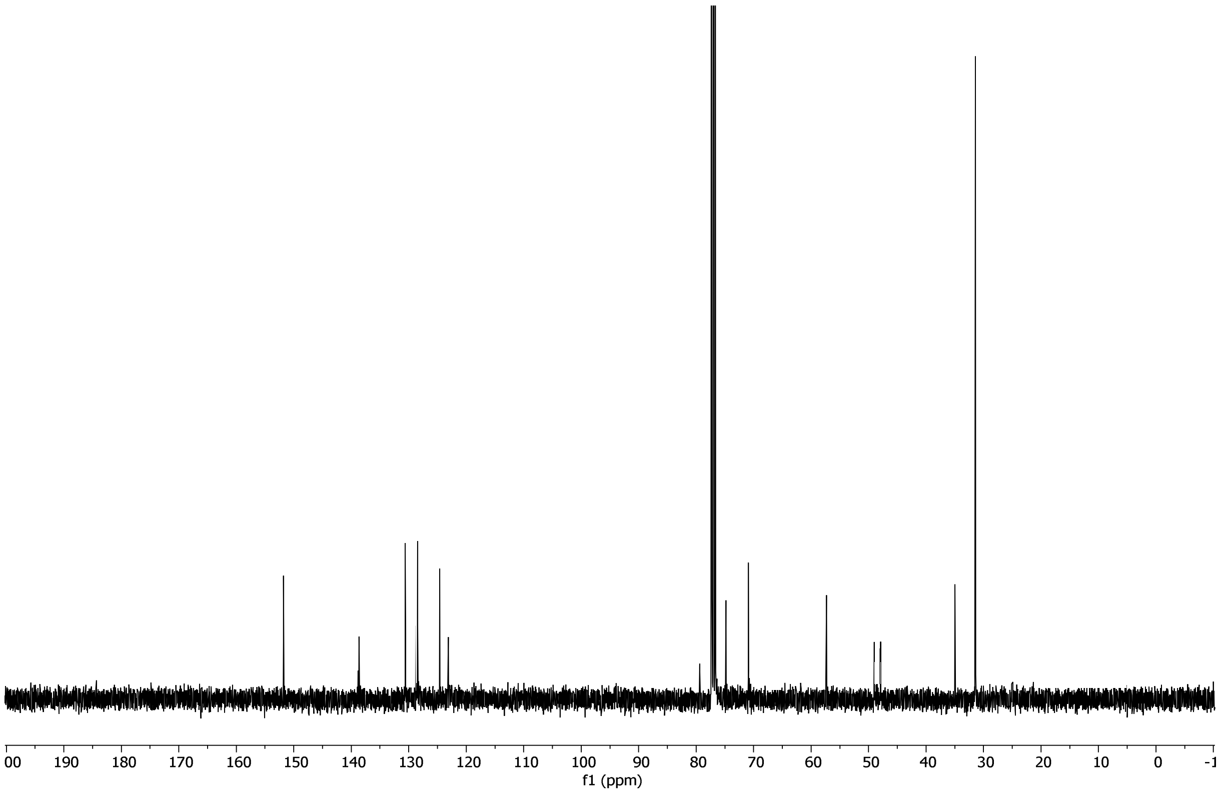

Supplementary Figure 20. ^{13}C NMR spectrum (100 MHz, $CDCl_3$) of **13**.

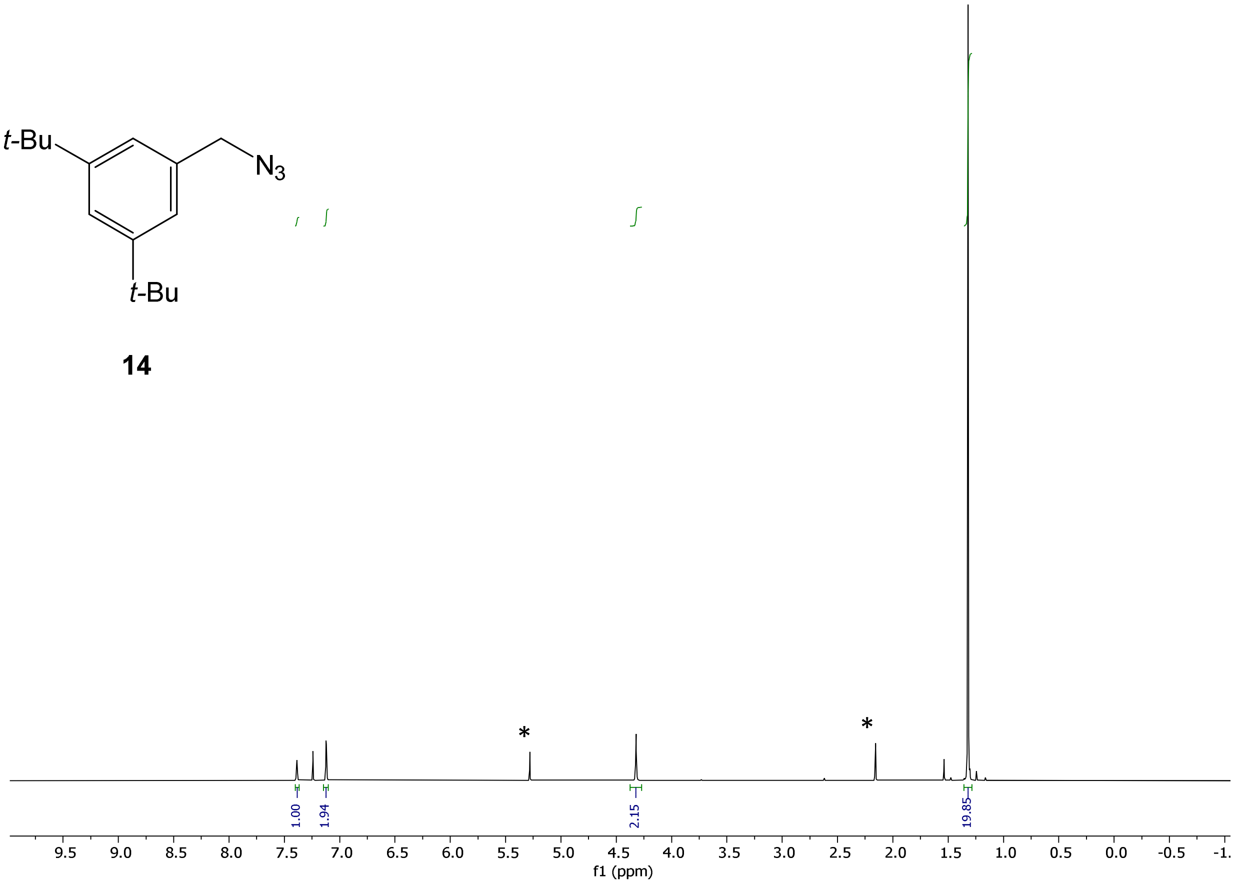

Supplementary Figure 21. ^{1}H NMR spectrum (400 MHz, $CDCl_3$) of **14**. Stars indicate residual solvents.

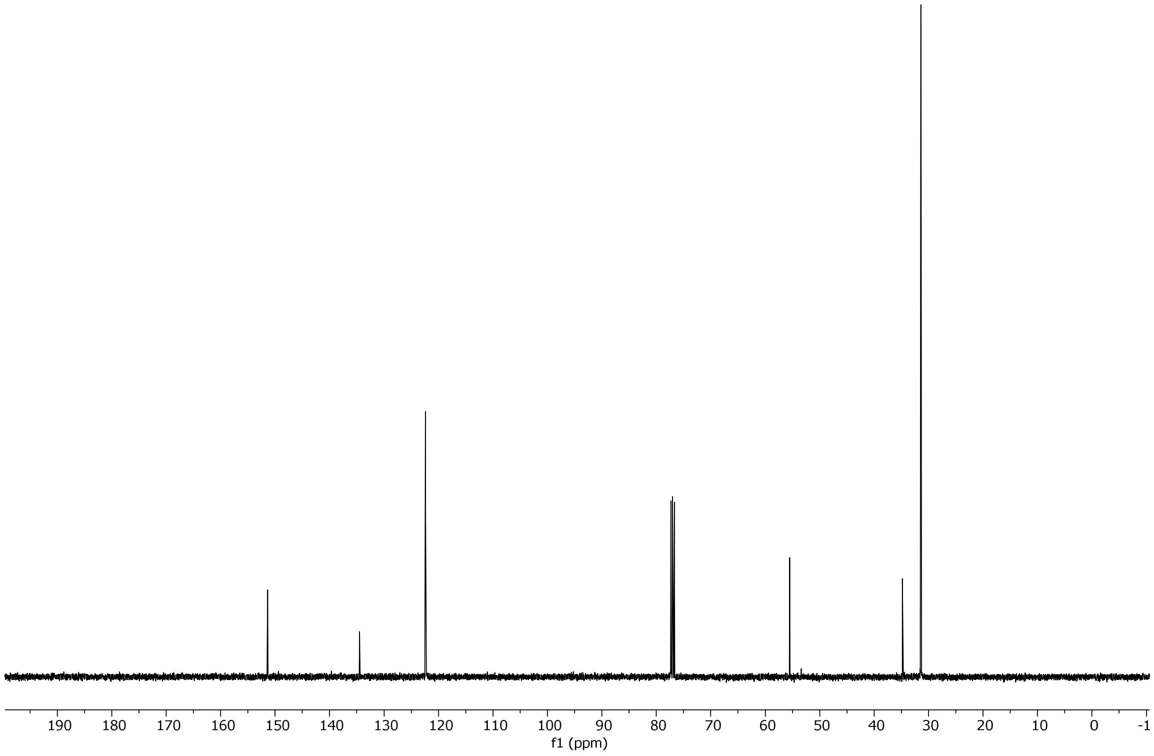

Supplementary Figure 22. ^{13}C NMR spectrum (100 MHz, $CDCl_3$) of **14**.

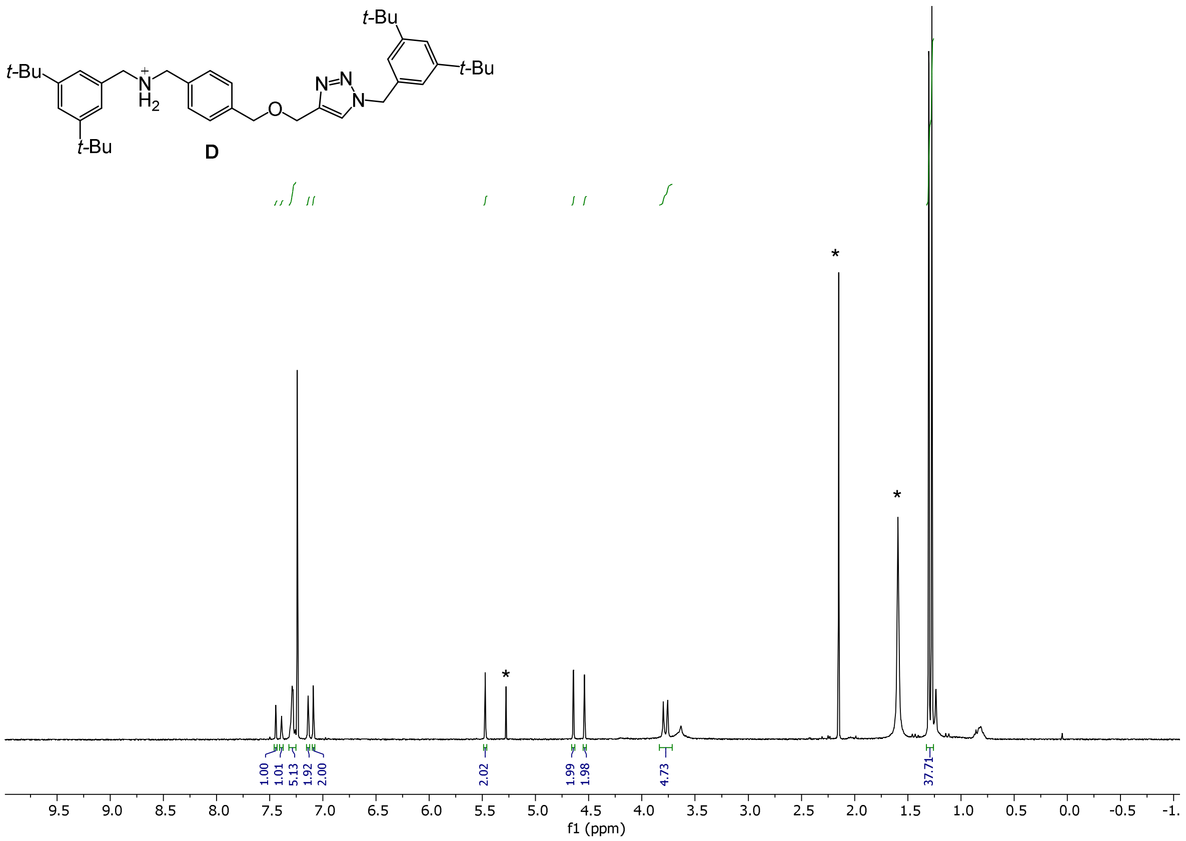

Supplementary Figure 23. ^{1}H NMR spectrum (400 MHz, $CDCl_3$) of **D**. Stars indicate residual solvents.

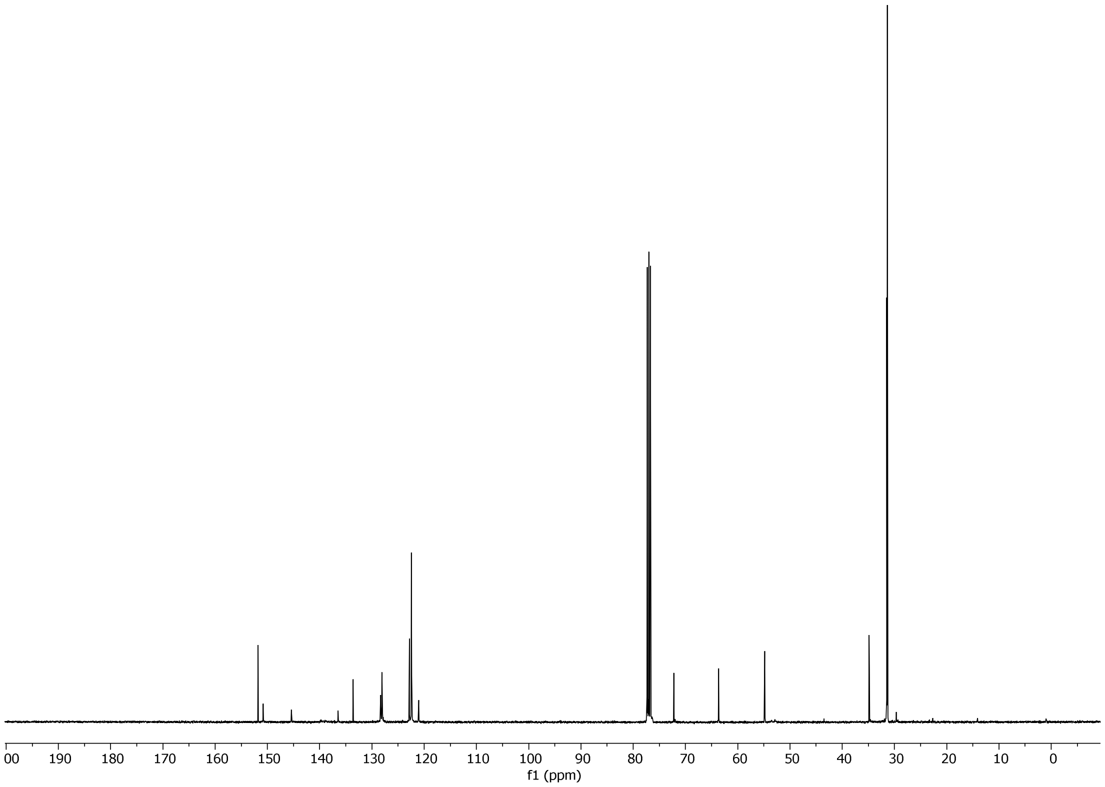

Supplementary Figure 24. ^{13}C NMR spectrum (100 MHz, $CDCl_3$) of **D**.

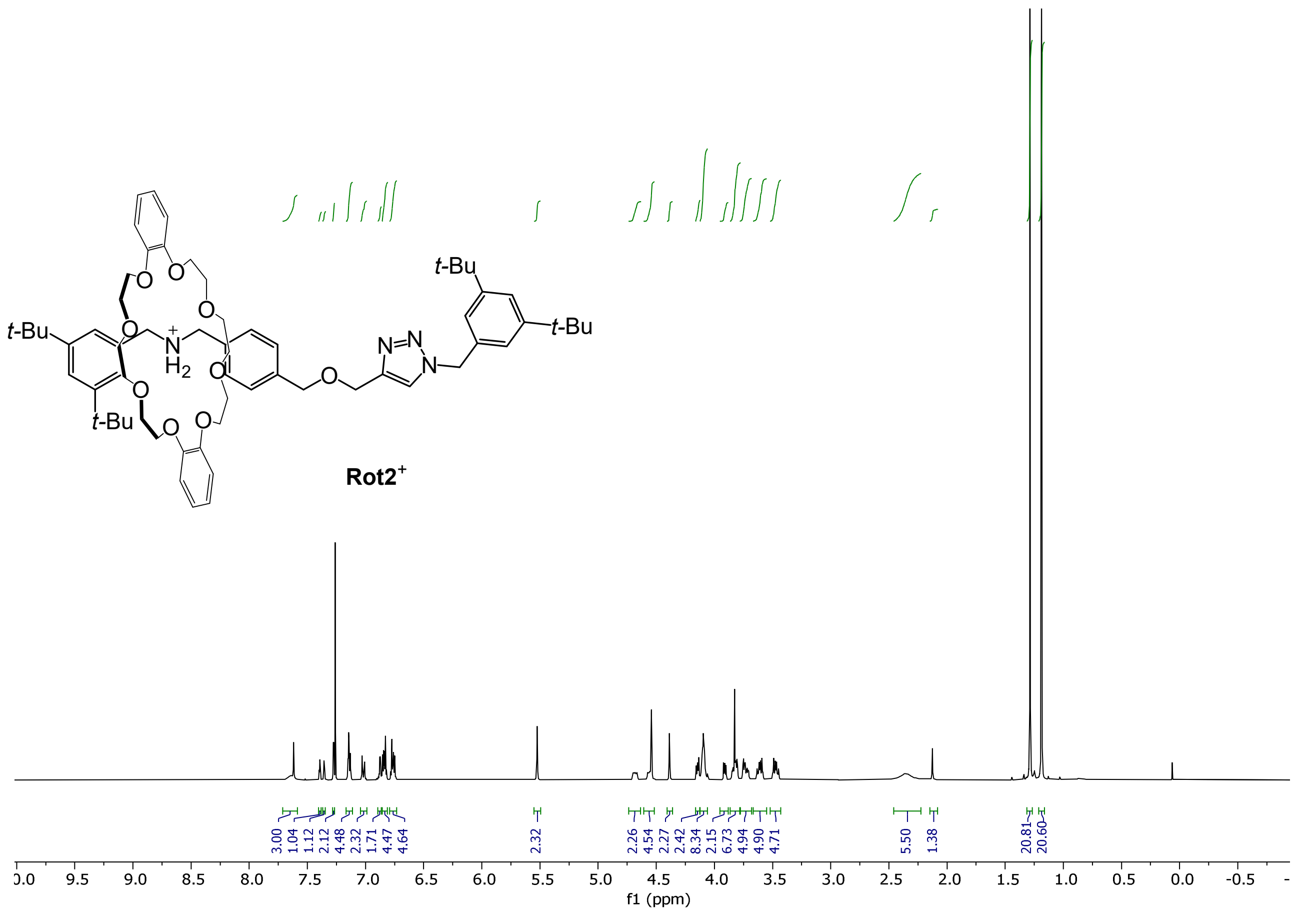

Supplementary Figure 25. ^{1}H NMR spectrum (400 MHz, $CDCl_3$) of **Rot2**$^{+}$.

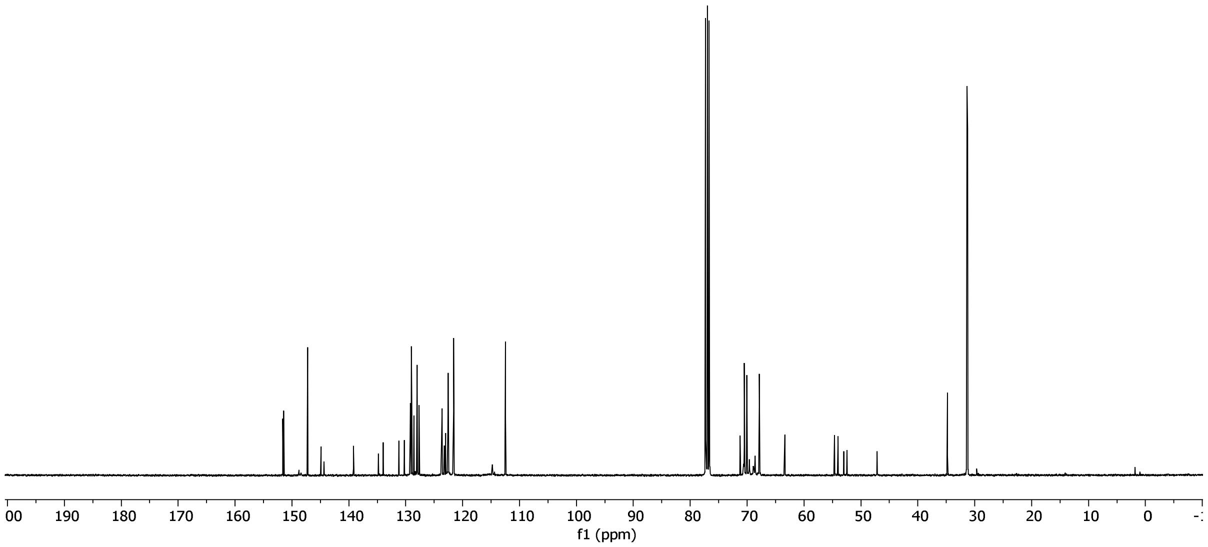

Supplementary Figure 26. ^{13}C NMR spectrum (100 MHz, $CDCl_3$) of **Rot2**$^{+}$.

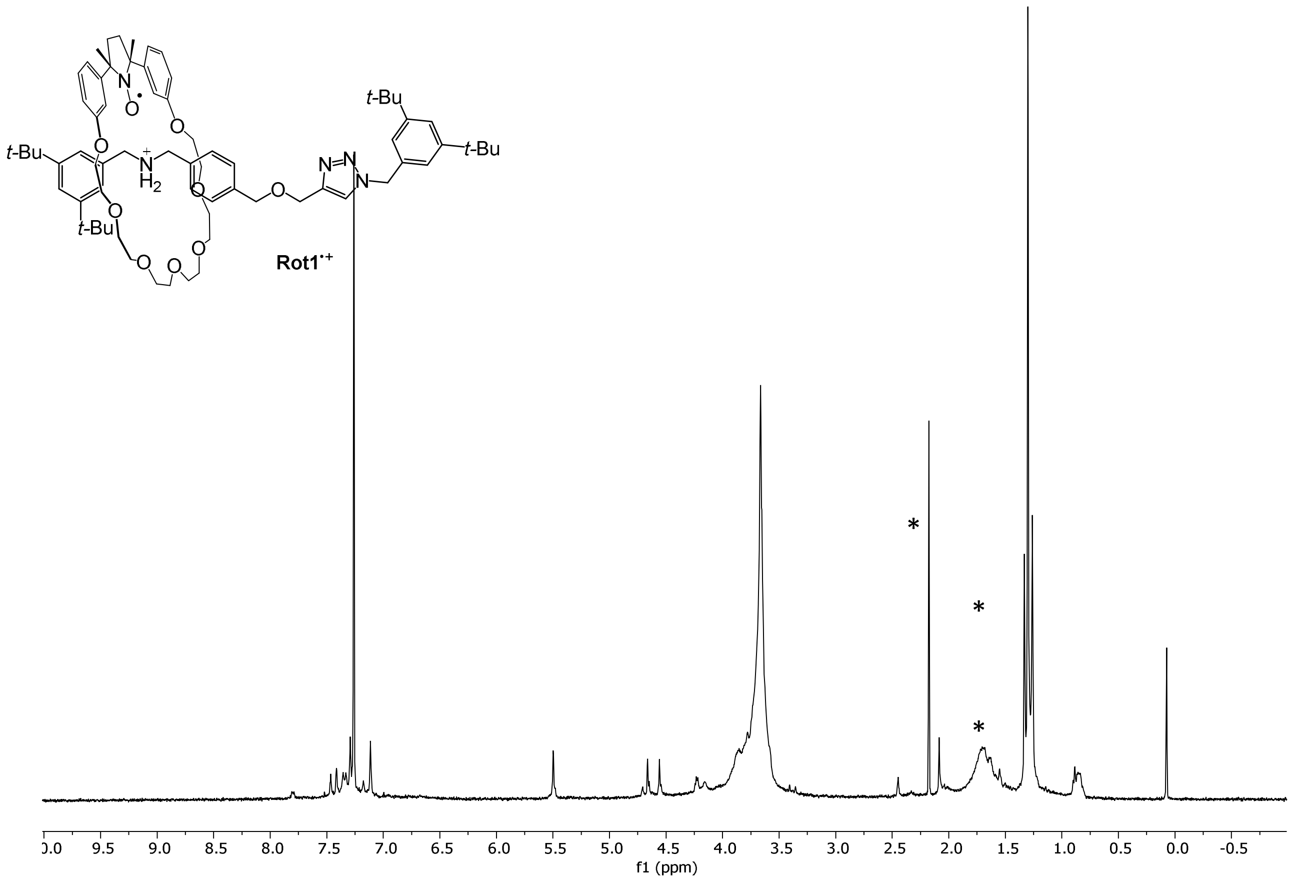

Supplementary Figure 27. ^{1}H NMR spectrum (400 MHz, $CDCl_3$) of **Rot1**$^{+\bullet}$. Stars indicate residual solvents.

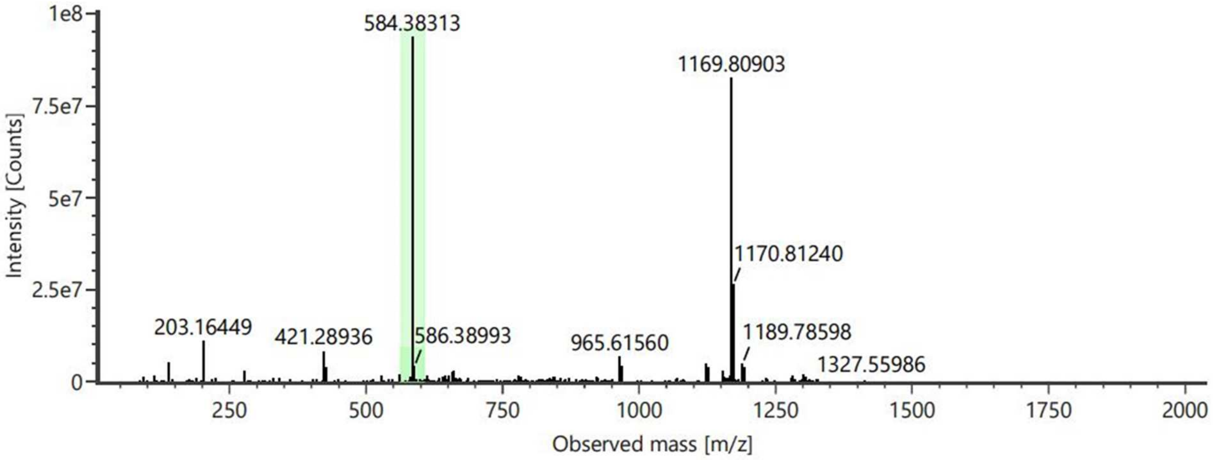

Supplementary Figure 28. HRMS spectrum of **Rot1**$^{+\bullet}$

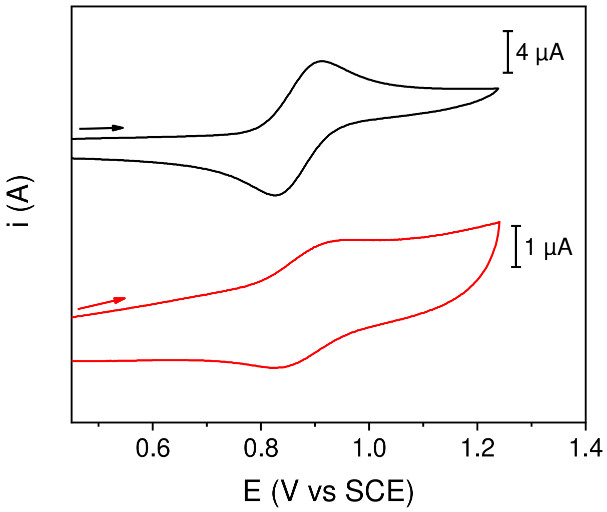

Supplementary Figure 29. Cyclic voltammograms of a CH_2Cl_2 solution of *cis*-**1**$^{•}$ (black line, 4.0 × 10^{-4} M) and of **Rot1**$^{+•}$ (red line, 1.0 × 10^{-4} M). Experimental conditions: argon-purged CH_2Cl_2, room temperature, 100 equivalents of $TBAPF_6$, scan rate: 200 mV/s, decamethylferrocene (FcMe10) used as internal standard.

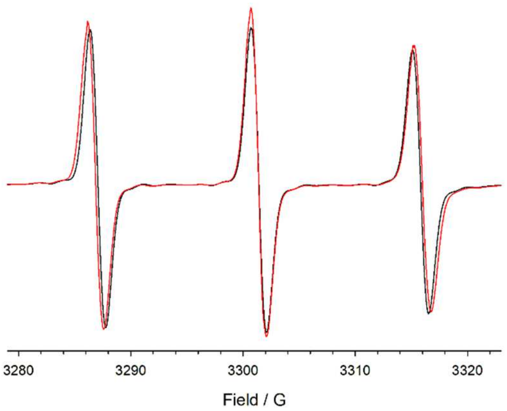

Supplementary Figure 30. Room temperature EPR spectrum of rotaxane **Rot1**$^{•+}$ in CH_3CN before (black line) and after sequential addition of P1-*t*-Bu (red line).

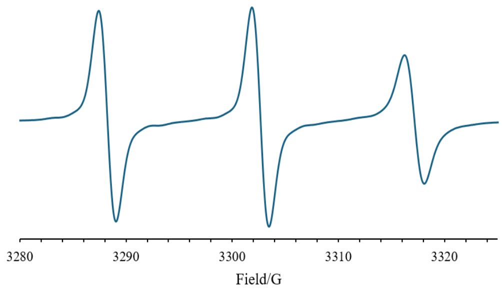

Supplementary Figure 31. Room temperature EPR spectrum of rotaxane **Rot1**$^{•+}$ 1.0 mM in DMSO. The spectrum shows a greater broadening of the third line due to the higher viscosity of the solvent itself.

3. Supplementary References

[1] Bleve, V. et al., *Chem. Eur. J.* **24**, 1198–1203 (2018).

[2] Cicchi, S.; Marradi, M.; Goti, A. & Brandi A. *Tetrahedron Lett.*, **42**, 6503–6505 (2001).

[3] Molander, G. A. & Shin, I. *Organic Lett.*, **15**, 2534-2537, (2013).

[4] Karaj, E. et al. S. *Bioorganic Chemistry*, **122**, 105700 (2022).

[5] Curcio, M.; Nicoli, F.; Paltrinieri, E.; Fois, F.; Tabacchi, G.; Cavallo, L.; Silvi, S.; Baroncini, M. & Credi, A. *J. Am. Chem. Soc.* **143**, 8046–8055 (2021).

[6] Campbell-Verduyn, L. Elsinga, P.H.; Mirfeizi, L.; Dierckx, R.A. & Feringa, B.L. *Org. Biomol. Chem.* **6,** 3461-3463 (2008).

[7] Treitler D. S. & Leung S. *J. Org. Chem.*, **87**, 11293–11295 (2022).
